# A distinct, high-affinity, alkaline phosphatase facilitates occupation of P-depleted environments by marine picocyanobacteria

**DOI:** 10.1073/pnas.2312892121

**Published:** 2024-05-07

**Authors:** Alberto Torcello-Requena, Andrew R. J. Murphy, Ian D. E. A. Lidbury, Frances D. Pitt, Richard Stark, Andrew D. Millard, Richard J. Puxty, Yin Chen, David J. Scanlan

**Affiliations:** ^a^School of Life Sciences, University of Warwick, Coventry CV4 7AL, United Kingdom; ^b^Molecular Microbiology: Biochemistry to Disease, School of Biosciences, University of Sheffield, Sheffield S10 2TN, United Kingdom; ^c^Centre for Phage Research, Department of Genetics and Genome Biology, University of Leicester, Leicester LE1 7RH, United Kingdom; ^d^School of Biosciences, University of Birmingham, Birmingham B15 2TT, United Kingdom

**Keywords:** phosphatase, *Synechococcus*, *Prochlorococcus*, phosphorus limitation, *psip1*

## Abstract

Marine picocyanobacteria of the genera *Prochlorococcus* and *Synechococcus*, the two most abundant phototrophs on Earth, thrive in oligotrophic oceanic regions. While it is well known that specific lineages are exquisitely adapted to prevailing in situ light and temperature regimes, much less is known of the molecular machinery required to facilitate occupancy of these low-nutrient environments. Here, we describe a hitherto unknown alkaline phosphatase, Psip1, that has a substantially higher affinity for phosphomonoesters than other well-known phosphatases like PhoA, PhoX, or PhoD and is restricted to clade III *Synechococcus* and a subset of high light I-adapted *Prochlorococcus* strains, suggesting niche specificity. We demonstrate that Psip1 has undergone convergent evolution with PhoX, requiring both iron and calcium for activity and likely possessing identical key residues around the active site, despite generally very low sequence homology. Interrogation of metagenomes and transcriptomes from TARA oceans and an Atlantic Meridional transect shows that *psip1* is abundant and highly expressed in picocyanobacterial populations from the Mediterranean Sea and north Atlantic gyre, regions well recognized to be phosphorus (P)-deplete. Together, this identifies *psip1* as an important oligotrophy-specific gene for P recycling in these organisms. Furthermore, *psip1* is not restricted to picocyanobacteria and is abundant and highly transcribed in some α-proteobacteria and eukaryotic algae, suggesting that such a high-affinity phosphatase is important across the microbial taxonomic world to occupy low-P environments.

## Significance

Marine picocyanobacteria are globally important primary producers, a facet facilitated via their ability to proliferate in nutrient impoverished regions of the sunlit ocean including oligotrophic gyres that are expected to expand due to climate change. Phosphorus is a major macronutrient potentially limiting growth and CO_2_ fixation capacity in such systems. Here, we identify a unique high-affinity phosphatase which in picocyanobacteria is present only in populations that occupy these P-deplete systems. This phosphatase is abundant and highly expressed in these regions, suggesting that genetic capacity exists within these populations to provide resilience to long-term P depletion. Moreover, this phosphatase is widely distributed in both heterotrophic bacteria and eukaryotic algae hinting that such a trait is broadly utilized to access such environments.

Picocyanobacteria are the most abundant group of marine phototrophs in the global ocean, dominated by two genera *Prochlorococcus* and *Synechococcus* (1–4) that contribute ~25% of total ocean primary production (5). While *Prochlorococcus* and *Synechococcus* shared a common ancestor approximately 823 to 644 Mya (6–8), they have significantly diverged in both a genomic (5, 9–11) and ecological context. In terms of biogeography, *Prochlorococcus* mainly dominates tropical and subtropical oceanic environments between ~45°N and 40°S (12–15), whereas *Synechococcus* has a more global distribution, occupying even polar waters (5, 15–17). This distribution requires that *Prochlorococcus* and *Synechococcus* can proliferate across a variety of environmental niches encompassing strong in situ gradients of light and temperature (4, 13, 17–19) and including the development of mechanisms to thrive in environments that are depleted in essential nutrients such as phosphorus (P).

These mechanisms can be in the form of high-affinity transporters for inorganic phosphate (Pi) (20–22). Alternatively, the ability to replace phospholipids with sulfolipids as a P-saving strategy (23) and fine-tuning their P-sensing and regulatory mechanisms can facilitate these two important processes (24, 25). Another key strategy for *Prochlorococcus* and *Synechococcus* to overcome low in situ P concentrations is the ability to utilize the plethora of organic P sources available in marine systems (26). Scavenging organic P largely revolves around the use of alkaline phosphatases (APases), hydrolytic enzymes that are present in diverse microorganisms (27) and which function to remineralize Pi largely from phospho-monoesters but in some cases phospho-diesters and -triesters (28), that represent available forms of dissolved organic P compounds in the ocean (29, 30).

APases, including PhoA, PhoD, and PhoX, typically differ in their metal requirements and/or substrate specificity (28, 31–37) or in the case of PafA are constitutive rather than low-P-inducible enzymes (27). These enzymes are also diverse in their cellular location, being found in the cytoplasm, the periplasm, as well as attached to the cell surface or secreted into the extracellular milieu (34). Across all bacteria, it was previously thought that *phoX* and *phoD* were more abundant in the oceans than *phoA* (33, 34), but more recent analyses show that both *phoA* and *pafA* are more prevalent than *phoX* in surface seawaters across the global ocean (27).

The abundance of P acquisition genes is known to vary according to local Pi concentrations in *Prochlorococcus* populations (22, 38), a feature that has been used to determine global-scale patterns of ocean nutrient limitation (39). However, few APases have been characterized in these organisms, apart from a PhoX type in a marine *Synechococcus* (35), despite the occurrence of several putative APases encoded in their genomes (11, 40). Occupation of P-depleted waters by the marine *Synechococcus* genus is largely facilitated by the presence of specific clades or ecologically significant taxonomic units (18) with members of *Synechococcus* clade III being especially well adapted to P-deplete oligotrophic waters given the number of niche-specific genes that appear to be P related (40). One such niche-specific gene is *psip1* encoding a phosphate starvation inducible polypeptide of unknown function (41, 42). Subsequent genomic analysis showed that *psip1* was indeed restricted to just a few HLI *Prochlorococcus* strains (e.g., *Prochlorococcus* sp. EQPAC1 and *Prochlorococcus* sp. MED4) and all clade III marine *Synechococcus* strains but no others (11). Analysis of P regulatory mechanisms in marine picocyanobacteria showed *psip1* to be regulated both by the PhoBR two-component system (25) and the PtrA transcriptional regulator, the latter a CRP (cyclic AMP receptor protein) family protein (24, 43). In *Synechococcus* sp. WH8102, PtrA up-regulates several genes in response to severe P stress, including putative alkaline phosphatases, a potential phytase, and various hypothetical genes including *psip1* (24). The fact that *ptrA* is regulated by the PhoBR two-component system suggests a transcriptional cascade response to P limitation/starvation with the first level mediated by the PhoBR system inducing the expression of high-affinity Pi transporter genes like *pstS* and the second regulated by *ptrA* likely a response to chronic P starvation (24).

Here, we show that *psip1* encodes a high-affinity alkaline phosphatase requiring calcium and iron for activity that is highly expressed in picocyanobacterial populations inhabiting P-deplete oceanic regions. Psip1 represents a distinct alkaline phosphatase family that is also present in α-proteobacteria and some eukaryotic phytoplankton suggesting the broad utility of such a function to inhabit a low-P niche across an extensive taxonomic front. Strikingly, despite no overall sequence identity, Psip1 shows structural similarity to the PhoX phosphatase with evidence for convergent evolution given that amino acid residues known to form part of the metal binding/active site are shared between the two proteins. Characterization of this single hypothetical protein among the myriad of known unknown proteins present in databases has thus not only identified an ecologically important niche-specific gene but also potentially opens a door to biochemically and structurally interrogate the basis of substrate affinity differences in this ecologically important group of phosphatase enzymes.

## Results and Discussion

### Psip1 Is a High-Affinity Alkaline Phosphatase.

**Fig. 1.**
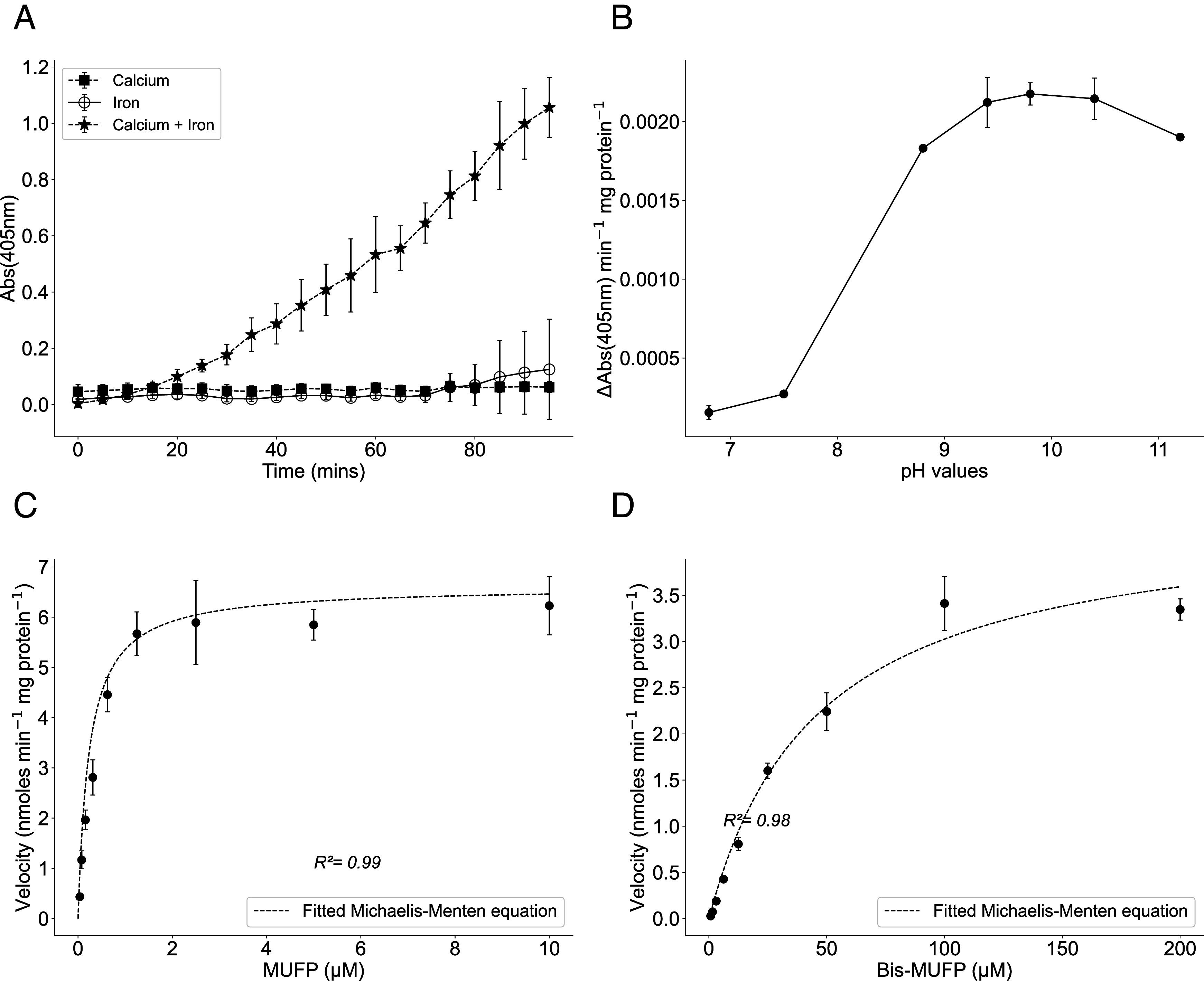
Biochemical characterization of Psip1 from *Prochlorococcus* sp. MED4. (*A*) Calcium and iron metal cofactors are required for Psip1 activity (Fe 0.1 mM; calcium 100 mM) (n = 6). (*B*) pH optimum for Psip1 activity. *p*NPP hydrolysis assays were carried out at different pH with Fe (0.1 mM) and Ca (100 mM) (n = 3). (*C*) Michaelis–Menten kinetics of Psip1 activity for MUF-P was carried by fitting the Michaelis–Menten equation to the V values (nmoles min^−1^ mg protein^−1^). (*D*) Michaelis–Menten kinetics of Psip1 activity for bis-MUF-P was carried by fitting the Michaelis–Menten equation to the V values (nmoles min^−1^ mg protein^−1^). Curve fitting was carried using Scipy 1.10.1 (n = 3).

Since *psip1* was previously known to be up-regulated by PtrA alongside other genes annotated to encode APases (24), we hypothesized that Psip1 had a similar phosphatase function. To biochemically characterize the protein a His-tagged recombinant Psip1 from *Prochlorococcus* sp. MED4 was overexpressed in *Escherichia coli* (*SI Appendix*, Fig. S1) and purified. Given the known requirement of metal cofactors for APase activity (35, 44) metal ions present in the purified protein were initially removed using EDTA so that the precise metal requirements could be ascertained. A variety of metal ions (specifically Ca^2+^, Mg^2+^, Mn^2+^, and Co^2+^ at 10 mM or Fe^3+^ at 10 µM concentration) were assessed for their ability to elicit phosphatase activity. Metals were tested alone or in combination with calcium and using 20 mM Tris buffer pH 8.8 as the reaction buffer (*SI Appendix*, Fig. S2). Only when iron and calcium were added together did Psip1 show phosphatase activity (Fig. 1*A*). This metal requirement for Psip1 activity is interesting since this mirrors the situation for the PhoX phosphatase which also requires iron and calcium for activity (37). From an ecological perspective, the iron requirement of Psip1 is consistent with clade III *Synechococcus* and HLI *Prochlorococcus* ecotypes, to which *psip1* is restricted, not occupying low-iron/high-nutrient low-chlorophyll (HNLC) waters (18). Psip1 APase activity was highest between pH 9.4 and 10.4 (Fig. 1*B*) clearly demonstrating the alkaline nature of this enzyme.

Further biochemical characterization of Psip1 showed that this phosphatase could cleave both the phosphomonoester substrate MUF-P (Fig. 1*C*) as well as the diester Bis-MUF-P (Fig. 1*D*). Kinetic analyses of Psip1 showed a K_m_ and V_max_ for MUF-P of 0.35 μM ± 0.033 (SD) and 6.6 ± 0.16 nmoles min^−1^ mg protein ^−1^, respectively. This highlights Psip1 as a high-affinity APase compared to other alkaline phosphatase families from various bacteria that have been characterized previously, with, for example, the K_m_ for PhoX often in the region of 90 μM or higher (Table 1 and *SI Appendix*, Table S1). Note that K_m_ and V_max_ values for the phosphodiester Bis-MUF-P, which were 46.1 ± 5.72 µM and 4.4 ± 0.21 nmoles min^−1^ mg protein^−1^, respectively, indicate that Psip1 has a substantially higher affinity for phosphomonoesters. Psip1 showed similar affinity for the phosphomonoester substrate *p*NPP with a K_m_ and V_max_ of 2.5 ± 1.18 μM and 11.7 ± 1.9 nmoles min^−1^ mg protein ^−1^ (*SI Appendix*, Fig. S3*A*), respectively, whereas no activity on the phosphodiester substrate Bis-*p*NPP was detected (*SI Appendix*, Fig. S3*B*). Finally, we estimated the affinity of Psip1 for a range of natural organic P compounds: glycerol-1-phosphate (G1P), glycerol-3-phosphate (G3P), phosphocholine (PC), phosphorylethanolamine (PE), adenosine monophosphate (AMP), and glucosamine-6-phosphate (Ga6P) by measuring the inhibition of MUF release from MUF-P when these organic P compounds are present (*SI Appendix*, Table S1 and Fig. S4). Although estimated K_i_ values (*SI Appendix*, Table S1) suggest slightly lower affinity than for MUF-P, our data nonetheless demonstrate greater affinity for these substrates compared to other classical APases. In order to determine that these organic compounds are substrates of Psip1 we also demonstrated Pi release using a phosphomolybdate assay (*SI Appendix*, Fig. S5), although, as this assay is less sensitive, we do not have equivalent kinetic data. We were not able to detect the inhibition of MUF release from Bis-MUF-P by the phosphodiester glycerophosphorylcholine (GPC), though Pi release from GPC was detectable (unlike from Bis-MUF-P) (*SI Appendix*, Fig. S4). Further functional characterization of Psip1 was precluded by our inability to obtain a fully segregated mutant of *psip1* in a marine *Synechococcus* and the lack of a genetic system for *Prochlorococcus*. Importantly, high-affinity APase activity has been previously measured in oceanic waters (e.g., see ref. 45). Hence, Psip1 possesses similarly low K_m_ values as these unidentified environmental APases.

**Table 1.** Comparison of K_m_ values between Psip1 and other known phosphatases

Protein	Organism	K_m_	Substrate	Optimal pH	Literature
Psip1					
	*Prochlorococcus* MED4	0.35 ± 0.03 μM	(MUF)-phosphate	8.8*	This work
		2.5 ± 1.18 μM	*p*NPP	9.8	
		46.10 ± 5.72	Bis-(MUF)-phosphate	8.8*	
PhoX					
	*Phaeobacter* sp. MED193	97 ± 10.3 μM	*p*-NPP	7.5*	(45)
		62.9 ± 11.3 μM	PC		
	*Sinorhizobium meliloti*	85.3 ± 4.5 μM	*p*-NPP	10*	(46)
		92.1 ± 8.7 μM	AMP		
	*Vibrio cholerae*	~240 μM	*p*-NPP	8.2	(47)
	*Pasteurella multocida* X-73	~95 μM	*p*-NPP	10*	(48)
PhoA					
	*Alteromonas mediterranea*	94 ± 35 μM	(MUF)-phosphate	8.2*	(28)
PhoD					
	*Aphanothece halophytica*	~3.38 mM	*p*-NPP	10*	(36)
	*Cobetia amphilecti*	~4.2 mM	*p*-NPP	9.2	(49)

*The K_m_ was obtained at the reported pH (not necessarily the optimal pH for enzyme activity).

Substrates: para-nitrophenylphosphate, *p*-NPP; phosphocholine, PC; adenosine monophosphate, AMP; 4-methylumbelliferyl phosphate, (MUF)-phosphate (*SI Appendix*, Table S1 for an extended version).

### Psip1 Represents a Discrete APase Family but with Predicted Structural Homology to PhoX.

**Fig. 2.**
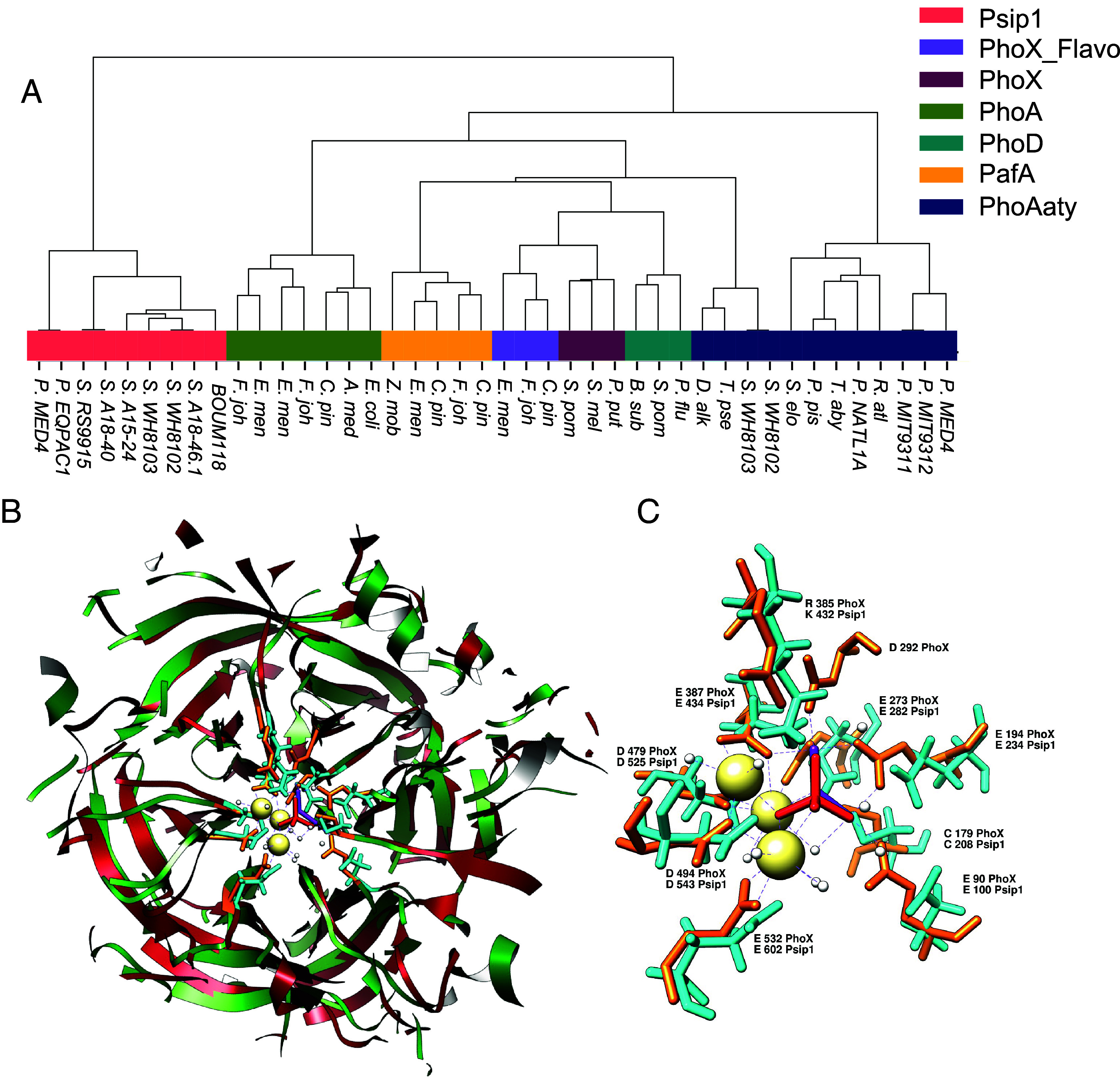
(*A*) Hierarchical clustering comparison between Psip1 and other major phosphatases. The dendrogram was built based on distances of e-values using reciprocal BlastP comparison between the sequences followed by hierarchical agglomerative clustering. Abbreviations: *F. joh, Flavobacterium johnsoniae; C. pin, Chitinophaga pinensis; E. men, Elizabethkingia meningoseptica; R. pom, Ruegeria pomeroyi; B. sub, Bacillus subtilis; P. flu, Pseudomonas fluorescens; P. put, Pseudomonas putida; Z. mob, Zymomonas mobilis; S. mel, Sinorhizobium meliloti; A. med, A. mediterranea*; P. MED4, *Prochlorococcus* sp. MED4; P. EQPAC1, *Prochlorococcus* sp. EQPAC1; P. MIT9311, *Prochlorococcus* sp. MIT9311; P. MIT9312, *Prochlorococcus* sp. MIT9312; P. NATL1A, *Prochlorococcus* sp. NATL1A; S. A18-46.1, *Synechococcus* sp. A18-46.1; S. WH8102, *Synechococcus* sp. WH8102; S. WH8103, *Synechococcus* sp. WH8103; S. A15-24, *Synechococcus* sp. A15-24; S. BOUM118, *Synechococcus* sp. BOUM118; S. A18-40, *Synechococcus* sp. A18-40; S. RS9915, *Synechococcus* sp. RS9915; *R. atl*, *Ruegeria atlantica*; *T. aby*, *Thalassobius abyssi*; *P. pis, Phaeobacter piscinae*; *S. elo, Synechococcus elongatus*; *T. pse, Thalassiosira pseudonana*; *D. alk, Desulfoglaeba alkanexedens*. (*B*) Structural analysis using UCSF-Chimera comparing Psip1 from *Prochlorococcus* sp. MED4 (red) with PhoX (green) from *P. fluorescens*. Overlapping of both proteins’ structures and alignment of their sequences revealed that the only element they share is the β-propeller structure. (*C*) PhoX amino acid residues indicated in orange are part of the metal-binding site forming a scaffold with calcium (yellow) and iron (purple) metal ions to bind the phosphoryl group (red). These amino acid residues are also conserved in Psip1 (in blue) at a similar spatial position.

Given the biochemical characterization of Psip1 as an APase above, we next examined its relationship to previously characterized members of this group of enzymes. BLASTP searches using Psip1 from *Prochlorococcus* sp. MED4 (PMM1416) only showed hits to proteins annotated as hypothetical or a (hemolysin-type) calcium-binding protein, while CDD analysis showed no conserved domains. This lack of sequence identity to known APases precluded a “normal” phylogenetics approach to deciphering the relationship of these proteins. Instead, we compared PhoA, PhoX, PhoD, PafA, and Psip1 protein sequences through reciprocal BLASTP and then clustered them using agglomerative clustering to assess differences more clearly among them. The resulting dendrogram showed that Psip1 is clearly distinct from other known phosphatases (Fig. 2*A*).

In contrast, structural homology modeling using Phyre2 (using Psip1 from *Prochlorococcus* sp. MED4) showed a match to the PhoX APase from *P. fluorescens* with confidence and coverage values of 99% and 72%, respectively. The model produced by Phyre2 was similar to one produced by AlphaFold. Based on analysis of the AlphaFold model using UCSF Chimera (*SI Appendix*, Fig. S6*A*), Psip1 and PhoX both possess a ß-propeller and a funnel-like structure formed by several ß-sheets (Fig. 2*B*). This domain is formed by six-bladed ß-sheets forming a putative active site. PhoX from *P*. *fluorescens* contains three calcium atoms and one iron atom as metal cofactors per native complex, forming a scaffold that binds to the phosphoryl group. Interestingly, amino acid residues that form part of the metal binding/active site of *P*. *fluorescens* PhoX are almost entirely shared by Psip1 (with similar spatial distribution) (Fig. 2*C*), and these amino acid residues show conservation when aligning Psip1 sequences from other marine picocyanobacteria (*SI Appendix*, Fig. S7). Two exceptions here are that an arginine residue at position 385 in PhoX is replaced with a functionally similar lysine residue in Psip1 (K432), while D292 in PhoX lacks a corresponding residue in Psip1 (from *Prochlorococcus* sp. MED4) or its function is carried out by another amino acid that does not align similarly in 3D space.

AlphaFold analysis revealed a clear signal peptide that was confirmed by SignalP3.0 and SignalP5.0 programs in Psip1 from *Prochlorococcus* sp. MED4 (*SI Appendix*, Fig. S6*B*) that seems to be absent in all *Synechococcus* Psip1 versions from currently sequenced genomes (*SI Appendix*, Fig. S7). This is consistent with previous experimental work that located Psip1 to the cell wall in *Prochlorococcus* (42). However, whether this location is the same for the *Synechococcus* Psip1, which lacks a signal sequence, remains to be determined. However, results from SecretomeP-2.0 strongly indicate that all Psip1 sequences from marine *Synechococcus* and *Prochlorococcus* are potentially secreted (SecP score > 0.9 in all sequences, min threshold is 0.5). Certainly, for other APases both cellular location and secretion machinery can differ. Thus, PhoA is often located to the periplasm and secreted via the Sec pathway (51) while PhoD, with a tendency to be located to the cytoplasm, and PhoX more extracellularly located have both been predicted to be secreted via the twin-arginine translocation (tat) pathway (33–36, 52). Together, our data suggest that Psip1 and PhoX may represent an example of convergent evolution in the APase family, although Psip1 shows much higher affinity for APase substrates.

### Marine Picocyanobacterial *psip1* Is Highly Expressed in Low-P Environments.

**Fig. 3.**
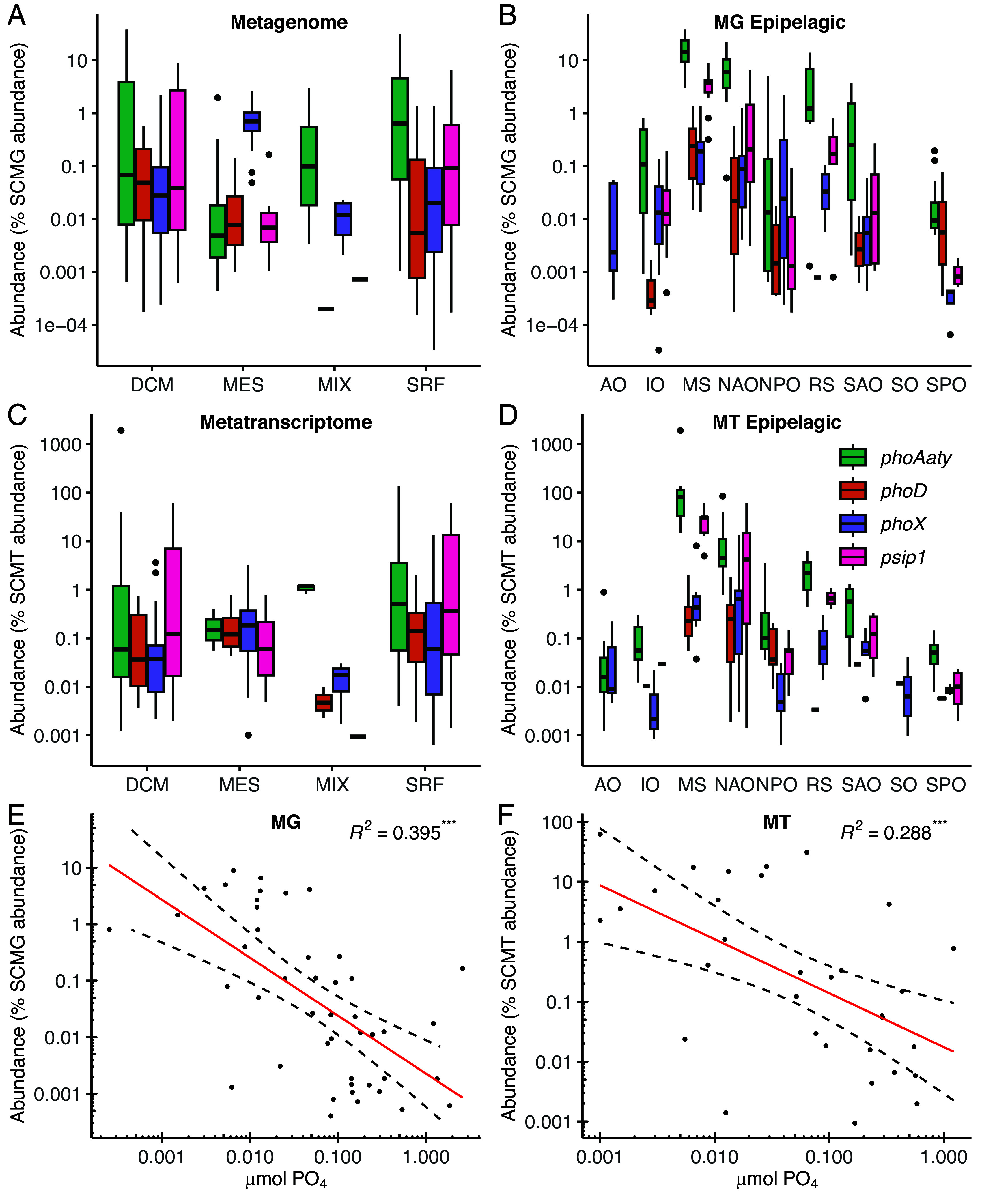
Expression of known cyanobacterial phosphatase genes compared to *psip1* in the TARA Oceans dataset. (*A*) The relative abundance of cyanobacterial APase genes in metagenomes, split by depth, expressed as a percentage of whole-community single-copy marker gene (SCMG) abundance. (*B*) The relative abundance of cyanobacterial APase transcripts in metatranscriptomes, split by depth, expressed as a percentage of whole-community single-copy marker transcript (SCMT) abundance. (*C*) The relative abundance of cyanobacterial APases in metagenomes split by oceanic region. (*D*) The relative abundance of cyanobacterial APase genes in metatranscriptomes split by oceanic region. For each boxplot, median values are shown with a horizontal black line, lower and upper hinges show the first and third quartiles, respectively, and whiskers extend to the largest/smallest value no larger/smaller than 1.5* the interquartile range outside the third and first quartiles, respectively. Outliers outside this range are shown as black dots. Abbreviations: MG, metagenome; MT, metatranscriptome; DCM, deep chlorophyll maximum; MES, mesopelagic; MIX, wind mixed layer; SRF, surface; AO, Arctic Ocean; IO, Indian Ocean; MS, Mediterranean Sea; NAO, North Atlantic Ocean; NPO, North Pacific Ocean; RS, Red Sea; SAO, South Atlantic Ocean; SO, Southern Ocean; SPO, South Pacific Ocean. (*E*) Correlations between the abundance of *psip1* and phosphate in the metagenome and (*F*) metatranscriptome of the TARA Oceans prokaryotic database. Linear regressions of log_10_ transformed abundance vs PO_4_ concentration are shown (red lines) together with 95% CI (dashed lines). R^2^ values are shown, ***P < 0.001.

We next sought to assess the environmental relevance of *psip1* using the TARA Oceans database. Noncyanobacterial sequences were filtered and excluded and the abundance/expression values from metatranscriptome and metagenome screens were normalized using a set of ten single-copy marker genes (53). The abundance of *psip1* as well as known picocyanobacterial APase genes (*phoX* and *phoD*) was also analyzed. We were unable to detect cyanobacterial *phoA* sequences despite previous studies reporting Pi-responsive *phoA* genes in marine *Prochlorococcus* (22, 38). Analysis of the putative *phoA* gene of *Prochlorococcus* sp. MED4 (22) revealed it to be a distant homolog (48% coverage, 30.8% identity, 6e-12) of an “atypical PhoA” (hereafter PhoAaty) first identified as an APase in freshwater *Synechococcus* but which bears no sequence similarity to the classical PhoA (54). Divergent PhoAaty proteins from photosynthetic eukaryotes have also demonstrated APase activity (55); we thus include *phoAaty* in our abundance analysis as a putative APase. Across TARA sampling types *psip1* was significantly more abundant within surface (SRF) as compared to mesopelagic (MES) waters (Fig. 3*A* and *SI Appendix*, Table S2). When comparing the ocean region, while there was generally no significant difference in *psip1* gene abundance compared to *phoX*/*phoD,* there was a clear exception in the Mediterranean Sea, where *psip1* gene abundance was significantly higher (Fig. 3*B* and *SI Appendix*, Table S3). Similarly, *psip1* showed significantly greater transcription than both *phoX* and *phoD* in the Mediterranean Sea (Fig. 3*D* and *SI Appendix*, Table S3). Full details of the statistical comparisons used for all APases can be found in Supplementary Information; we note that the putative *phoAaty* is also abundant within many of the regions with high *psip1* gene/transcript abundance (Fig. 3). These patterns of *psip1* gene and transcript abundance agree well with another genetic adaptation to chronic P stress, membrane lipid remodeling, which is also known to be widespread in microbial assemblages in the Mediterranean Sea and other P deplete oceanic regions (56). To confirm this pattern, we determined that across all sample sites, there are statistically significant negative relationships between log-transformed *psip1* abundance and Pi concentrations in both metagenomic and metatranscriptomic datasets (Fig. 3 *E* and *F*) (R^2^ = 0.395, *P* < 0.001, and R^2^ = 0.288, *P* < 0.001, respectively). Noteworthy, here, is that previous analysis of gene frequencies of P-related genes in *Prochlorococcus* populations from the Bermuda Atlantic Time Series (BATS, North Atlantic) and Hawaii Ocean Time Series (HOT, Pacific Ocean) observed a much higher number of *psip1* (PMM1416) reads at BATS compared to HOT (38), the former being a known P-depleted environment like the Mediterranean Sea (57, 58). Additionally, we used available TARA metadata to examine the relationship between *psip1* abundance and other environmental variables. Of note, there was a significant positive correlation between iron concentration and *psip1* relative abundance in both metagenomes (*rho* = 0.57, *P* < 0.001) and metatranscriptomes (*rho* = 0.59, *P* < 0.001). Given the iron requirement of Psip1 for enzymatic activity, it stands to reason that where iron is scarce, *psip1* relative abundance is low. Several other environmental parameters correlated with *psip1* abundance (*SI Appendix*, Table S4). However, many of these parameters co-correlated with other environmental parameters. Thus, we used linear regression models to control for co-correlation. Using these analyses, only PO_4_ concentration had a significant negative correlation with *psip1* relative abundance in metagenomes (*t* = −7.62, *P* < 0.001) and metatranscriptomes (*t* = −2.74, *P* = 0.009). Conversely, NO_3_ concentration had a significant positive correlation with *psip1* in both metagenomes (*t* = 2.99, *P* = 0.004) and metatranscriptomes (*t* = 2.15, *P* < 0.05). Thus, when PO_4_ concentration is low, increases in NO_3_ concentration may lead to exacerbated P stress due to N:P stoichiometry and therefore increase selection on *psip1* relative abundance and/or expression.

**Fig. 4.**
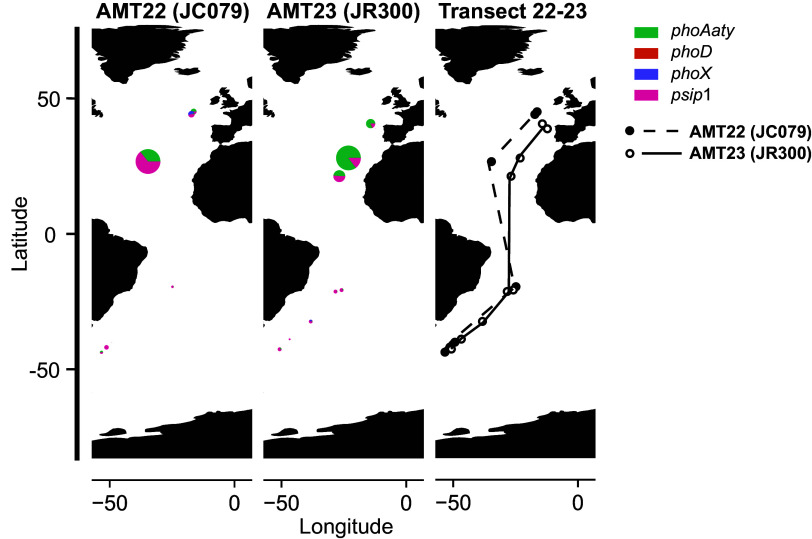
The expression of *psip1* along an Atlantic Meridional Transect in 2012 (AMT22) and 2013 (AMT23) compared to other cyanobacterial phosphatases. The *Left* two panels indicate the level of expression of the different phosphatases, while the *Right* panel indicates the location of the stations sampled in each cruise. The level of expression in each station is indicated by the size of the pie chart. While expression of *phoX* and *phoD* was detected in these samples, *psip1* and *phoAaty* expression covered the majority of the reads in stations from the North Atlantic Ocean.

Furthermore, when we examined our own metatranscriptomics datasets obtained from Atlantic Meridional Transects undertaken in 2012 and 2013 (AMT22 and AMT23), again focusing solely on picocyanobacterial sequences, *psip1* and *phoAaty* had much higher expression values compared to *phoX* and *phoD*, despite the fact that their relative abundance seems to shift between the two datasets. This was particularly evident at two stations in the North Atlantic gyre AMT22_18_18 and AMT23_11_15 (Fig. 4 and *SI Appendix*, Table S5). Taken together, these data demonstrate high expression of *psip1* in low-P environments across the world’s oceans suggesting an important role for this enigmatic gene. Moreover, when we compared *psip1* gene abundance with the nutrient stress genes used by Ustick et al. (39) in their “biosensor” strategy to infer nutrient limitation in *Prochlorococcus*, *psip1* had a different distribution across TARA stations, as shown by nMDS ordination of gene abundances (*SI Appendix*, Fig. S8). Thus, *psip1* may be an improved marker for phosphate stress than those previously used (39) but which requires further characterized genes like *psip1* of the high P stress severity type to substantiate this.

### *psip1* Was Horizontally Transferred between Functionally and Phylogenetically Distinct Taxa.

The presence of *psip1* in marine picocyanobacteria is restricted to marine *Synechococcus* (clade III) and some HLI *Prochlorococcus* (e.g., MED4 and EQPAC1). Members of *Synechococcus* clade III are well described as preferentially occupying P-depleted oligotrophic waters (18, 59) and hence carriage of *psip1* specifically in these strains is consistent with this being a niche-specific gene. In *Prochlorococcus* sp. MED4 *psip1* (PMM1416) is found in a specific genomic island associated with other P-starvation-expressed genes (21, 41). In contrast, *psip1* in *Synechococcus* clade III is not located in a predicted genomic island. Such a difference likely highlights the “assimilation” of *psip1* into the clade III *Synechococcus* genomic backbone which has become a prerequisite for their occupation of oligotrophic low-P niche waters. In contrast, only members of the more broadly distributed HLI *Prochlorococcus* ecotype occupying P-deplete waters seem to have acquired *psip1* and appear to have done so via horizontal transfer of a genomic island (41).

**Fig. 5.**
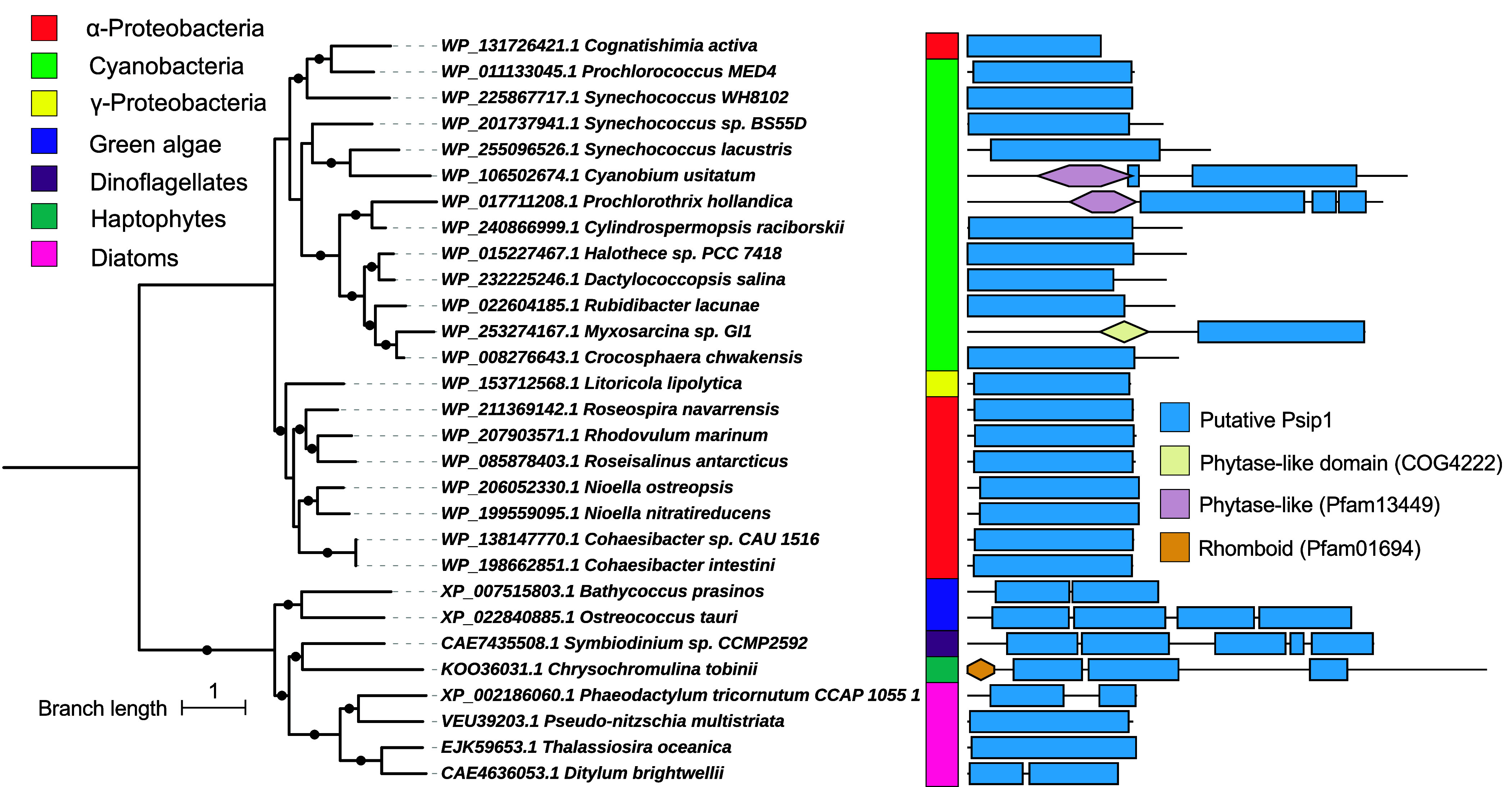
Psip1 phylogeny. The phylogenetic tree was created from BlastP hits using the RefSeq database (e-value < 10^−20^). The alignment was manually curated. The Psip1-like domain was created using sequences from marine cyanobacteria and hmmbuild and then using hmmsearch in the sequences to confirm the hits. Other domains were obtained using CDDsearch (e-value < 10^−20^) and then plotted along the sequence alongside the Psip1-like domain. Bootstrap values > 70 are indicated with a black dot.

Given the potential horizontal transfer of *psip1* into some HLI *Prochlorococcus* populations we sought to determine the origin of this gene and hence more broadly define the taxonomic breadth of organisms within which *psip1* resides. To do this a BLASTP search was carried out using a strict e-value cutoff (1e-20) and an alignment of the output sequences used to build a profile HMM for *psip1*, which we subsequently used to again scrutinize the TARA Oceans database. Sequences retrieved from this search were then added to those recovered by BLASTP and a new alignment manually curated before further phylogenetic analysis was performed (*SI Appendix*, Fig. S9). Psip1 ORFs were present in a wide variety of taxa beyond picocyanobacteria including heterotrophic bacteria mostly from the α-proteobacteria, e.g., *Roseospira navarrensis*, *Nioella ostreopsis*, and *Cognatishimia activa*, but also eukaryotic phytoplankton including diatoms and green algae (Fig. 5). Interestingly, a domain search of Psip1 orthologs in other organisms showed that the Psip1 domain can also be accompanied by other functional domains, e.g., in *Cyanobium usitatum*, *Prochlorothrix hollandica*, and *Myxosarcina* sp. GI1 (ß cyanobacteria) that have an additional phytase-like domain, hinting at the potential to produce functional diversity within this phosphatase family beyond that just associated with Psip1. In addition, some eukaryotic ORFs contain multiple Psip1 domains, e.g., the green algae *Bathylococcus prasinus* and *Ostreococcus tauri*, the dinoflagellate *Symbiodinium* sp. CCMP2592, and the haptophyte *Chrysochromulina tobinii*.

### Eukaryotic *psip1* Homologs Are Also Highly Transcribed in P-Deplete Oceanic Regions.

**Fig. 6.**
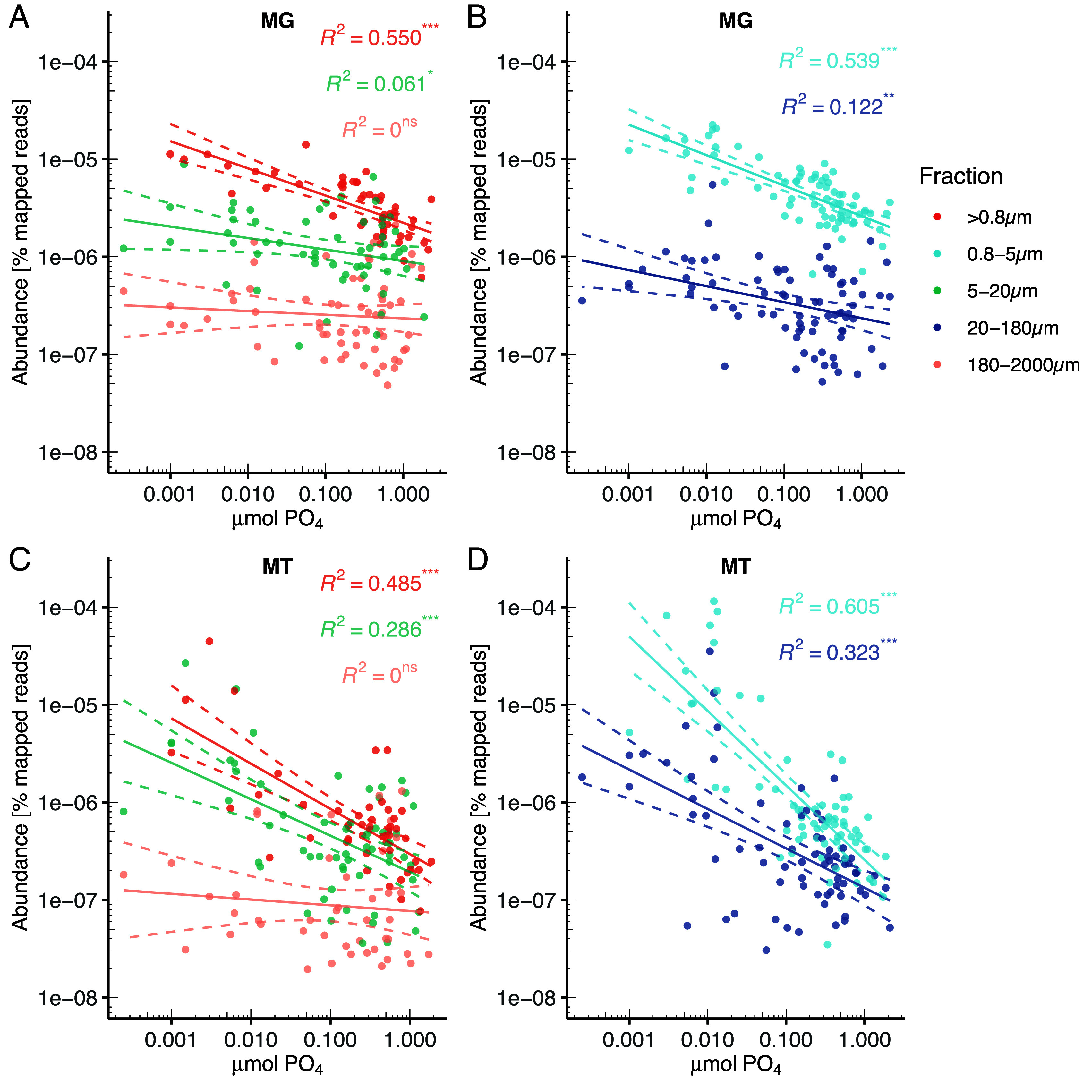
Correlation between the abundance of *psip1* and inorganic phosphate (PO_4_) concentrations in the metagenome (*A* and *B*) and metatranscriptome (*C* and *D*) of the Marine Atlas TARA Ocean Unigenes (MATOU) database. Abundance values are split according to the size fraction applied prior to nucleic acid extraction. Fractions are split into panels for clarity of interpretation. Linear regressions of log_10_ transformed abundance vs. PO_4_ concentration are shown (solid lines) together with 95% CI (dashed lines). R^2^ values are shown in the corresponding color. *P < 0.05, **P < 0.01, ***P < 0.001, ns = not significant.

In order to assess the environmental abundance and transcription of these eukaryotic *psip1* homologs, and their relationship with standing stock Pi concentrations, we used the TARA Oceans Marine Atlas of TARA Oceans Unigenes (MATOU) database (60). Abundance data (expressed as a percentage of mapped reads) were split into fractions based on the size filters applied to samples. Within the MATOU metagenome, *psip1* abundance again showed a significant negative relationship with Pi concentration in the >0.8, 0.8 to 5, 5 to 20, and 20 to 180 µm fractions, but not in the 180 to 2,000 µm fraction (Fig. 6 *A* and *B*). Within the MATOU metatranscriptome, the same pattern was repeated (Fig. 6 *C* and *D*), suggesting that *psip1* is both more abundant in the genomes of, and more highly transcribed by, photosynthetic eukaryotes living in Pi deplete regions, consistent with a role in releasing Pi from organic P sources in these organisms. To compare *psip1* with other phosphatases within photosynthetic eukaryotes, we repeated these analyses on *phoA*, *phoD*, *phoAaty*, and *phoX* sequences from the MATOU databases. Interestingly, no significant negative relationship between Pi concentration and abundance was found for either *phoA* or *phoD*, in either metagenomes or metatranscriptomes (*SI Appendix*, Fig. S10 *C–F*)—indeed both were significantly more abundant in high Pi regions in some fractions of the metagenome (but not metatranscriptome) dataset (*SI Appendix*, Fig. S10). While the metagenomic abundance of *phoAaty* showed no relationship with Pi concentration, transcription showed significant negative relationships with Pi concentration in the >0.8 and 0.8 to 5 µm fractions (*SI Appendix*, Fig. S10*A*). Significant negative relationships with Pi concentration and *phoX* abundance were found within the >0.8 and 0.8 to 5 µm fractions of the metagenome (*SI Appendix*, Fig. S10*B*), whereas significant negative relationships with Pi concentration and *phoX* transcription were found within the >0.8, 0.8 to 5, 5 to 20, and 20 to 180 µm fractions, but not in the 180 to 2,000 µm fraction (*SI Appendix*, Fig. S10 *G* and *H*). The phylogenetic relationship between environmental MATOU *psip1* sequences and those found in genome-sequenced organisms is shown in *SI Appendix*, Fig. S11, together with total oceanic abundance of each sequence within the MATOU metatranscriptome across all sites within each fraction (*SI Appendix*, Fig. S11). Broad taxonomic assignment, expressed as a percentage of transcripts from a given fraction, differs significantly between fractions (Χ^2^ = 241, *P* <0.001), with Holm’s corrected pairwise comparisons showing significant differences between all fractions with the exception of >0.8 and 0.8 to 5 µm, and 5 to 20 and 180 to 2,000 µm (*SI Appendix*, Table S6). Both >0.8 and 0.8 to 5 µm fractions contain a large percentage of unclassified sequences (45% and 46%, respectively), with haptophytes and dinoflagellates making up most of the remainder (34% and 14%, and 29% and 13%, respectively) (*SI Appendix*, Table S6). Both 5 to 20 and 180 to 2,000 µm show a relatively even split between haptophyte, dinoflagellate, and diatom transcripts (26%, 32%, and 37%, and 39%, 23%, and 27%, respectively), while the 20 to 180 µm fraction is dominated by diatom transcripts (75%). These data provide insight into the classes of phosphatase that photosynthetic eukaryotes use to acquire P from organic sources in their environment.

## Conclusions

Psip1 is a distinct, high-affinity APase with a K_m_ value in the low micromolar range, a feature entirely compatible with the observed low organic P concentrations found in P-deplete oligotrophic gyre regions (61) occupied by clade III *Synechococcus* and some high light *Prochlorococcus* strains. Indeed, molecular ecological data show *psip1* to be highly expressed in marine picocyanobacteria occupying such low-P environments, especially compared to other described phosphatases, suggesting that this is an important niche-specific gene. The presence of *psip1* in heterotrophic bacterial taxa as well as eukaryotic phytoplankton hints at a broader utility of such a function. Furthermore, regulation of *psip1* by the CRP family regulator PtrA (24), which in turn is regulated by the PhoBR two-component system (25), is consistent with the biochemical properties of Psip1 and maximal expression of its gene in low-P oligotrophic environments. Indeed, such a hierarchical regulatory mechanism is well established in bacterial utilization of nitrogen, which is probably best studied in nitrogen fixation, e.g., the two-component system NtrBC regulating the expression of NifA that is specifically required for the transcription of nitrogenase (62). Together, our results suggest that PtrA acts as a regulator of a hyperefficient P starvation response that induces phosphatases like Psip1 with extremely high affinity for a range of organic P substrates, highlighting the exquisite nature of how microbes adapt to their real-world environment.

## Materials and Methods

### Heterologous Overexpression of Psip1 in *E*. *coli*.

Psip1 from *Prochlorococcus* sp. MED4 was used for overexpression in *E*. *coli*. The predicted signal peptide (using SignalP 3.0 and SignalP 5.0) (63, 64) was removed, and the nucleotide sequence codon optimized for *E*. *coli*, prior to cloning into pET-23a (+), which attaches a His-Tag at the C terminus of the protein. Overexpression was in *E*. *coli* BL21(DE3) cells grown in LB at 37 °C until an OD_600_ of 0.4 was reached. Cultures were then induced with 0.5 mM IPTG and incubated for 25 h with shaking at 180 rpm using a gradually decreasing temperature program as follows: 1 h at 37 °C, 2 h at 30 °C, and 18 h at 22 °C. Cells were harvested by centrifugation, and cell pellets were immediately frozen in liquid nitrogen and stored at −20 °C for future purification.

### Purification of Psip1.

Cell samples from the Psip1 overexpression were resuspended in 50 mL lysis buffer (10 mM HEPES, 250 mM NaCl, 0.5 mM TCEP, 5% Glycerol, 1 tablet of cOmplete™ Protease Inhibitor Cocktail, and 1X BugBuster® Protein Extraction Reagent) and then lysed using a One-Shot Cell disrupter (Constant Systems) 2× at 20 kilopounds per square inch (kpsi). Lysed samples were collected by centrifugation at 2880×*g* for 10 min at 4 °C (Eppendorf Centrifuge 5810R) to separate the soluble and insoluble fractions. The soluble supernatant fraction was collected and filtered through a 0.22 µm pore-size Whatman® Puradisc filter (GE Healthcare). Filtered lysate was purified using a nickel-affinity purification column (Roche). Protein concentration was measured using the Bradford assay (65). To purify Psip1 from inclusion bodies, the protocol of Palmer and Wingfield was used (66). Briefly, inclusion bodies were washed three times in 100 mM Tris, pH 7.5, containing 2 M Urea, 1% v/v Triton X-100, and 5 mM EDTA, prior to being washed twice in 100 mM Tris, pH 7.5, containing 5 mM Urea. Inclusion bodies were then solubilized in 50 mM Tris, pH 7.5, containing 8 M Guanidine hydrochloride, and dialyzed overnight in 10 mM Tris, pH 7.5, containing 250 mM NaCl, 10 mM CaCl_2_ and 1 µM FeCl_3_.

### Determination of Metal Cofactors for Psip1.

To assess the metal cofactor requirement of Psip1, it was first necessary to remove any metals already bound to the protein during purification. To achieve this Psip1 samples were treated with 50 mM EDTA, pH 6.8 in loading buffer (20 mM Tris/HCl pH 8, 250 mM NaCl, 0.5 mM TCEP) at 4 °C overnight. To remove the EDTA a PD-10 desalting column protocol was used. The following trace metal cofactors were assessed: calcium (II), magnesium (II), manganese (II), iron (III), and zinc (II). Each metal stock, apart from iron, was treated using Chelex 100 Resin (Bio-Rad®) to remove any potential traces of iron that may be carried while preparing them. We assessed each metal either alone or in combination with calcium. All metals were used at 10 mM concentration except iron (10 µM).

### Alkaline Phosphatase Assays.

APase activity was assessed using the artificial substrates methylumbelliferyl-phosphate (MUF-P) and *p*-nitrophenyl phosphate (*p*NPP). Phosphatase hydrolysis of MUF-P releases the fluorescent compound methylumbelliferone (MUF) which was detected using an excitation wavelength of 360 nm and an emission wavelength of 460 nm, while phosphatase hydrolysis of *p*NPP releases the yellow-colored product *p*-nitrophenol (*p*NP), which was measured at an absorbance of 405 nm. Negative controls used either no protein sample or no substrate. Calf intestinal alkaline phosphatase (Promega) was used as a positive control. All conditions and controls were tested in triplicate at 30 °C for 5 h. Assays were carried out in 96-well plates and analyzed using either a FLUOstar Omega (BMG Labtech) plate reader, or a Nano+ (Tecan Life Sciences) plate reader. Phosphatase assays were conducted over a 3 h period, with measurements taken every 2 min. Activity curves were fitted using Python package Scipy 1.10.1 (67) to obtain the change in absorbance at 405 nm per minute. Phosphodiesterase activity was assessed using bis-*p*-nitrophenyl phosphate (bis-*p*NPP), the degradation of which also releases *p*NP, or bis-methylumbelliferyl-phosphate (bis-MUF-P), the degradation of which also releases MUF. *F. johnsoniae*, which show phosphodiesterase activity (68), was used as a positive control. To assess the optimal pH for enzyme activity, we compared Psip1 activity across a pH range between 6.8 and 11.2 in the presence of 0.1 mM iron (III) and 100 mM calcium.

Michaelis–Menten constants (V_max_ and K_m_) for the MUF-P substrate were obtained using 10, 5, 2.5, 1.25, 0.625, 0.3125, 0.15625, and 0.078125 µM concentrations of MUF-P in reaction buffer comprising 0.1 mM iron (III), 10 mM Ca^2+^, and 20 mM Tris-HCl pH 8.8. The MUF calibration curve used 2.34, 4.69, 9.38, 18.75, 37.5, 75 pmoles standards. Controls followed the same procedure, and all conditions and controls were repeated in triplicate. Fluorescence was corrected using the standard curve to estimate nmoles per minute, and the rate of reaction normalized to the amount of protein used. Rates were measured every 3 min and curves fitted to the linear part of the reaction. Reaction rates (nmoles/min/mg protein) were plotted against MUF-P substrate concentration. After plotting, the Michaelis–Menten curve was fitted using the equation: V_max_ * ([S]/(Km + [S])). K_m_ and V_max_ values were obtained using Python package Scipy 1.10.1 (67).

Determination of Pi liberation from organic P compounds was carried out following the protocol of Lidbury et al. (68). Briefly, 50 µL of enzyme reaction was added to 50 µL dH_2_O:6N sulfuric acid:2.5% w/v ammonium molybdate:20% sodium ascorbate in a 5:2:2:1 ratio and incubated at 37 °C for 1 h prior to measurement at 820 nm using a Fluostar Omega microplate reader. Absolute quantification of Pi was measured by comparison to a standard curve of K_2_HPO_4_.

### Structural Modeling of Psip1.

To initially characterize Psip1 we used Phyre2 (69), SwissModel (70), CDD Search (71, 72), and AlphaFold (73) through the Google Colab notebook (AlphaFold.ipynb—shorturl.at/asY06) using the default options. Visualization of Psip1 and structural analysis also used UCSF Chimera (74). Using templates provided by Phyre2 amino acid residues involved in metal-binding site were predicted, as well as potential signal peptides predicted by AlphaFold. Signal peptides were also assessed using SignalP 3.0 (63) and SignalP 5.0 (64). Potential secretion of Psip1 was assessed using SecretomeP-2.0 (75). Additionally, UCSF Chimera facilitated comparison of protein structures while MUSCLE v3.8.31 (76) was used to align protein sequences to identify conserved amino acids. When comparing Psip1 and PhoX structures, UCSF Chimera was used to overlap the structures and align the sequences.

### Evaluation of the Abundance and Expression of Psip1 in Marine Systems.

Two environmental datasets, AMT (https://www.amt-uk.org/) and TARA Oceans (77), were used to assess the abundance and expression of *psip1* in metagenomics and metatranscriptomics datasets. Data from AMT22 (JC079) and AMT23 (JR300) (78) provided information about the number of Reads Per Kilobase of transcript per Million mapped reads (RPKM) and the number of Transcripts Per Million (TPM). To query the TARA Oceans dataset, first BLASTP was used to extract all the psip1 sequences in the NCBI database, using an e-value threshold E > 1e-20. Sequences were aligned using MUSCLE v.3.8.31 (74), to be used as input for hmmbuild (hmmbuild {outputhmm} {inputalignment}) to create an HMM of Psip1 (79, 80). Finally, this was then used to query the TARA Oceans database (https://tara-oceans.mio.osupytheas.fr/ocean-gene-atlas/). Results were filtered based on e-value (1e-20) first, and then, only hits assigned within the cyanobacteria phylum in the TARA database were selected. With the final list, *psip1* gene abundance provided by the TARA dataset was normalized to the median abundance of ten single-copy housekeeping genes (from all bacteria) (53). Similarly, transcript abundance was normalized to the median transcript abundance of the same single-copy housekeeping genes (53). Finally, using R, results from these metatranscriptome and metagenome analyzed were plotted by TARA Oceans station and depth, as well as by ambient Pi concentrations. Pearson correlations between TARA metadata and log-transformed *psip1* relative abundance were performed in MATLAB. Significance testing was corrected using the Benjamini–Hochberg approach (81). Some independent variables were log transformed as shown in *SI Appendix*, Table S4. Variables that showed a significant correlation with *psip1* were subsequently used as predictor variables in a linear regression model as computed using the fitlm function in MATLAB (*SI Appendix*, Table S4).

To assess the abundance and expression of phosphatases within marine eukaryotic metagenomes and metatranscriptomes, the TARA Oceans MATOU (Marine Atlas of TARA Oceans Unigenes) databases were used. The aforementioned Psip1 HMM was used to query the databases using an e-value cutoff of 1e-20. Noneukaryotic sequences were discarded. Sequences were aligned with MED4 Psip1, and sequences shorter than 300 amino acids, or with greater than two gaps and/or one mismatch to the predicted metal ion binding sites (Fig. 2*C*) were discarded from further analysis. As all remaining sequences were found within photosynthetic clades, the abundance of other phosphatases within these photosynthetic clades was also examined. For PhoA and PhoD, HMMs were downloaded from pfam.xfam. However, for “atypical” PhoA (PhoAaty) and PhoX, characterized sequences from refs. 82–86 together with closely related sequences were aligned using ClustalOmega and HMMs were constructed using hmmbuild. This was necessary as PhoAaty has no defined HMM, and the existing PhoX HMM is entirely derived from bacterial sequences and was a poor match for the divergent characterized eukaryotic sequences (82, 86). Appropriate e-value thresholds for these HMMs were manually selected using hmmsearch on sequences from Haptophyta, Dinophyceae, Chlorophyta, and Bacillariophyta within UniprotKB. These thresholds were for PhoA, PhoD, PhoAaty, and PhoX, respectively, 1e-20, 1e-40, 1e-40, and 1e-100. Abundance data were collected as a percentage of mapped reads, with no further normalization. Sequences not belonging to the aforementioned photosynthetic eukaryotic groups were discarded from further analysis. Abundance data were split by filter size fractions, and fractions that were only present at few sites and/or localized to a single oceanic region were discarded from further analysis.

### Phylogenetic Analysis of Psip1.

Psip1 orthologs beyond marine picocyanobacteria were retrieved using BLASTP and a combination of the RefSeq and NCBI nonredundant protein databases using Psip1 from *Prochlorococcus* sp. MED4 as the query. An e-value of 1e-5 was used as a cutoff. Protein sequences were aligned using MUSCLE v3.8.31 (76) and the output manually curated to correct any potential errors or mismatches. The final alignment was used to build the phylogenetic tree using IQTREE v 1.6.3 (87), using ModelFinder (88) to select the best phylogenetic model for these data. The tree was subsequently annotated using iTOL (89).

To analyze domain structures, sequences retrieved above were subjected to a CDDsearch and the results exported as a table. The Psip1 HMM and the HMMsearch tool were used to identify the Psip1 domain. Using Python, domains identified using both the CDD and Psip1 HMMsearch outputs were combined and linked with the abovementioned tree.

Additionally, homologs of known APases (PhoX, PhoD, PhoA, and PafA) were compared with Psip1 by reciprocal BLASTP. e-values between groups were used to create a distance matrix between the different sequences. Distances were then used for Hierarchical agglomerative clustering using the Euclidean distances and “ward” method for clustering (90).

## Supplementary Material

Appendix 01 (PDF)

## Data Availability

The datasets generated during the current study are available as follows: Paired-end reads of the transcriptomics data obtained from AMT cruises JC079 and JC300 have been deposited in the European Nucleotide Archive (ENA) at EMBL-EBI under accession number PRJEB61548 (91). The computer code used in our analyses as well as hmm profiles, alignments, and protein sequences used in phylogenetic trees can be found in GitHub (https://github.com/Sechyss/Psip1_Data) (92). All other data are included in the manuscript and/or *SI Appendix*.
