# Supplementary material for: A distinct, high-affinity, alkaline phosphatase facilitates occupation of P-depleted environments by marine picocyanobacteria
Source: Proc Natl Acad Sci U S A. 2024 May 7;121(20):e2312892121. doi: 10.1073/pnas.2312892121 (PMC11098088; doi:10.1073/pnas.2312892121)
Supplement: Supplementary file 1 — Appendix 01 (PDF) [file pnas.2312892121.sapp.pdf]

Supplementary Information for:

A distinct, high affinity, alkaline phosphatase facilitates occupation of P-depleted environments by marine picocyanobacteria

Alberto Torcello-Requena[1], Andrew Murphy[1], Ian D. E. A. Lidbury[2], Frances D. Pitt[1], Richard Stark[1], Andrew D. Millard[3], Richard J. Puxty[1], Yin Chen[4], David J. Scanlan[1]

[1] School of Life Sciences, University of Warwick, Coventry, CV4 7AL, UK

[2] Molecular Microbiology: Biochemistry to Disease, School of Biosciences, University of Sheffield, Sheffield, UK

[3] Department of Genomics, University of Leicester, UK

[4] School of Biosciences, University of Birmingham, UK

Supplementary Analysis

Alkaline Phosphatase gene and transcript abundance across the Tara Oceans dataset

All APases showed significant differences in metagenome abundance by sampling site type (Kruskal-Wallis X^2 of 11.0 ($p<0.05$), 21.1 ($p<0.001$), 78.3 ($p<0.001$), and 10.6 ($p<0.05$) for *phoAaty*, *phoD*, *phoX*, and *psip1*, respectively) (Fig 3A). *phoAaty*, *phoD*, and *psip1* were significantly more abundant in SRF sampling sites, as compared to MES sites, and *phoAaty* and *phoD* were also significantly more abundant in DCM sites, whereas *phoX* was significantly more abundant in MES sites than in SRF or DCM sites (see Table S2 for full details of the Holm's corrected Dunn's test for multiple comparisons performed here). No significant difference could be detected between MIX sites and other categories, though there are only seven such sites, limiting the power of this analysis. Repeating this analysis showed significant differences in transcript abundance by site type for *phoAaty* and *phoX* (Kruskal-Wallis X^2 of 13.9 ($p<0.05$) and 32.8 ($p<0.001$) but not *phoD* or *psip1* (Fig 3C). Similarly, following post-hoc testing as above *phoAaty* showed significantly higher transcription in SRF and DCM sites, compared to MES sites, whereas *phoX* was significantly more abundant in MES sites than in SRF or DCM sites (Table S2). Again, no significant difference could be detected between MIX sites (seven in total), and other categories. All oceanic regions excluding the Southern Ocean showed significant differences in APase metagenome abundance (Fig 3B, Table S3) using the Kruskal Wallis test with outliers removed, though *psip1* was significantly more abundant than both *phoX* and *phoD* in the Mediterranean Sea, whereas *phoAaty* was also significantly more abundant than both *phoX* and *phoD* in the Indian Ocean, North Pacific, and South Atlantic regions (see Table S3 for full details of post-hoc testing). *psip1* transcription was significantly higher than both *phoX* and *phoD* in the Mediterranean Sea whilst *phoAaty* transcription was more abundant than both *phoX* and *phoD* in the Indian Ocean, Mediterranean Sea, and the North Atlantic (Fig. 3D, Table S3).

Supplementary Tables

Table S1. Reported K_m values and pH optima for different phosphatases from this work and previous literature.

**Alkaline phosphatase**	**Species**	**K_m (Substrate)***	**Optimum pH**	**Reference**
**Psip1**	*Prochlorococcus* sp. MED4	2.5 µM (*p*NPP)<br>0.35 µM (MUF-P)<br>46.1 µM (bis MUF-P)	9.8<br>8.8<br>8.8	This work
**PhoX**	*Sphingopyxis* sp. (metagenome)<br>*Sinorhizobium meliloti*<br>*Phaeobacter* sp. MED193<br>*Vibrio cholerae*<br>*Pasteurella multocida X-73*	180 µM (*p*NPP)<br>85.3 µM (*p*NPP), 92.1 µM (AMP)<br>97 µM (*p*NPP), 62 µM (PC)<br>240 µM (*p*NPP)<br>95 µM (*p*NPP)	9<br>10<br>7.5<br>8.2<br>10	(1)<br>(2)<br>(3)<br>(4)<br>(5)
**PhoA**	*Halomonas sp. 593*<br>*Pyrococcus abyssi*<br>*Shewanella sp. SIB1*<br>*Streptomyces griseus IMRU 3570*<br>*Thermotoga maritima*<br>*Vibrio G15-21*<br>*Alteromonas mediterranea*	ND**<br>166 µM (*p*NPP)<br>ND<br>130 µM (*p*NPP)<br>175 µM (*p*NPP)<br>170 µM (*p*NPP)<br>94 µM (MUF-P)	10.5<br>11<br>10.5<br>9.5<br>8<br>9<br>8.2	(6)<br>(7)<br>(8)<br>(9)<br>(10)<br>(11)<br>(12)
**PhoD**	*Antarctic bacterium HK47*<br>*Aphanothece halophytica*<br>*Cobetia amphilecti*	ND<br>3.38 mM (*p*NPP)<br>4.2 mM (*p*NPP)	9.5<br>10<br>9.2	(13)<br>(14)<br>(15)

* Substrates: para-nitrophenylphosphate, *p*NPP; phosphocholine, PC; adenosine monophosphate, AMP; 4-methylumbelliferyl phosphate, MUF-P; bis-methylumbelliferyl-phosphate (bis-MUF-P).

** ND – not determined

Table S1 cont. K_i values for Psip1 obtained using non-linear regression using the Michaelis Menten equation for competitive inhibition are given for different phospholipids and phosphosugars i.e. glycerol 3-phosphate (G3P), phosphorylethanolamine (PE), phosphocholine (PC), adenosine monophosphate (AMP), glucosamine-6-phosphate (Ga6P), and glycerol 1-phosphate (G1P). These values were estimated using the nls command in R. Km and Ki are µM. Vmax is nmoles/min/mg protein. Pr(>|t|) refers to the proportion of the t distribution greater than the t-value and is conceptually equivalent to a p value.

	**Estimate**	**Std. Error**	**t-value**	**Pr(>|t|)**	**Significance**
**G3P**					
K_m	0.65	0.09	7.37	2.85E-09	***
V_{max}	6.08	0.22	28.22	<2.0E-16	***
K_i	0.79	0.15	5.38	2.54E-06	***
**PE**					
K_m	0.68	0.09	7.85	5.65E-10	***
V_{max}	6.18	0.20	30.98	<2.0E-16	***
K_i	4.46	1.08	4.12	0.000161	***
**PC**					
K_m	0.61	0.07	8.54	5.66E-11	***
V_{max}	6.11	0.18	33.44	<2.0E-16	***
K_i	2.20	0.37	5.96	3.65E-07	***
**AMP**					
K_m	0.68	0.06	10.85	3.82E-14	***
V_{max}	6.35	0.15	41.97	<2.0E-16	***
K_i	1.52	0.22	6.79	2.06E-08	***
**Ga6P**					
K_m	0.29	0.04	7.70	2.85E-09	***
V_{max}	6.00	0.17	36.14	<2.0E-16	***
K_i	0.60	0.11	5.50	1.71E-06	***

**G1P**					
K_m	0.31	0.03	10.43	1.37E-13	***
V_{max}	6.20	0.12	49.71	<2.0E-16	***
K_i	1.20	0.20	5.97	3.43E-07	***

Significance codes: 0 ‘***’; 0.001 ‘**’; 0.01 ‘*’; 0.05 ‘.’; 0.1 ‘ ’ 1.

Table S2. Statistical comparisons of APase abundance within sampling sites and sampling site type across oceanic regions in the TARA Oceans dataset. Within each dataset, Kruskal Wallis test results followed by Holm's corrected post hoc Dunn's test for multiple comparisons are presented. These test results are also presented in an alternate format; each category is assigned a group by letter or letters. Categories sharing the same letter are not statistically different from one another. Abbreviations MG- metagenome; MT- metatranscriptomes; DCM- deep chlorophyll maxima; SRF- surface; MES- mesopelagic; MIX; wind mixed layer; AO- Arctic Ocean; IO- Indian Ocean; MS- Mediterranean Sea; NAO-North Atlantic Ocean; NPO- North Pacific Ocean; RS- Red Sea; SAO- South Atlantic Ocean; SO- Southern Ocean; SPO- South Pacific Ocean. Sheets are labelled to reflect the test being conducted; thus DCM_MG contains test data for comparisons between abundance of the four phosphatases within the DCM samples of the TARA metagenome (MG), whereas phoAaty_MG contains test data for comparisons between abundance of *phoAaty* across the sampling site types (DCM, SRF, MIX, and MES) within the TARA metagenome (MG).

1) DCM_MG

Kruskal Wallis Test

n	statistic	df	p
211	19.80301	3	0.000186

Dunn's Test (Holm's Correction)

Comparison	group1	group2	n1	n2	statistic	p	p.adj	p.adj.signif
phoAaty - phoD	phoAaty	phoD	53	52	-4.28299	1.84E-05	0.000111	***
phoAaty - phoX	phoAaty	phoX	53	53	-1.64845	0.099261	0.297783	ns
phoAaty - psip1	phoAaty	psip1	53	53	-2.84529	0.004437	0.022186	*
phoD - phoX	phoD	phoX	52	53	2.642409	0.008232	0.032927	*
phoD - psip1	phoD	psip1	52	53	1.451281	0.146702	0.297783	ns
phoX - psip1	phoX	psip1	53	53	-1.19684	0.231368	0.297783	ns

	Gene	Group
1	phoAaty	a
2	phoD	b
3	phoX	ac
4	psip1	bc

2) MES_MG

Kruskal Wallis Test

n	statistic	df	p
152	95.64036	3	1.34E-20

Dunn's Test (Holm's Correction)

Comparison	group1	group2	n1	n2	statistic	p	p.adj	p.adj.signif
phoAaty - phoD	phoAaty	phoD	38	38	1.382485	0.166823	0.179836	ns
phoAaty - phoX	phoAaty	phoX	38	38	7.473221	7.83E-14	3.91E-13	****
phoAaty - psip1	phoAaty	psip1	38	38	-1.69583	0.089918	0.179836	ns
phoD - phoX	phoD	phoX	38	38	6.090735	1.12E-09	4.50E-09	****
phoD - psip1	phoD	psip1	38	38	-3.07832	0.002082	0.006245	**
phoX - psip1	phoX	psip1	38	38	-9.16905	4.77E-20	2.86E-19	****

	Gene	Group
1	phoAaty	ab
2	phoD	a
3	phoX	c
4	psip1	b

3) MIX_MG

Kruskal Wallis Test

n	statistic	df	p
20	10.3045	3	0.0161

Dunn's Test (Holm's Correction)

Comparison	group1	group2	n1	n2	statistic	p	p.adj	p.adj.signif
phoAaty - phoD	phoAaty	phoD	5	5	-0.99467	0.319897	0.95969	ns
phoAaty - phoX	phoAaty	phoX	5	5	1.813811	0.069707	0.278827	ns
phoAaty - psip1	phoAaty	psip1	5	5	-0.93616	0.349191	0.95969	ns
phoD - phoX	phoD	phoX	5	5	2.808481	0.004978	0.029865	*
phoD - psip1	phoD	psip1	5	5	0.05851	0.953342	0.95969	ns
phoX - psip1	phoX	psip1	5	5	-2.74997	0.00596	0.029865	*

	Gene	Group
1	phoAaty	ab
2	phoD	a
3	phoX	b
4	psip1	a

4) SRF_MG

Kruskal Wallis Test

n	statistic	df	p
332	33.20608	3	2.91E-07

Dunn's Test (Holm's Correction)

Comparison	group1	group2	n1	n2	statistic	p	p.adj	p.adj.signif
phoAaty - phoD	phoAaty	phoD	83	83	-5.69463	1.24E-08	7.42E-08	****
phoAaty - phoX	phoAaty	phoX	83	83	-2.1449	0.031961	0.063922	ns
phoAaty - psip1	phoAaty	psip1	83	83	-2.89535	0.003787	0.015149	*
phoD - phoX	phoD	phoX	83	83	3.54973	0.000386	0.001928	**
phoD - psip1	phoD	psip1	83	83	2.799275	0.005122	0.015365	*
phoX - psip1	phoX	psip1	83	83	-0.75045	0.452981	0.452981	ns

	Gene	Group
1	phoAaty	a
2	phoD	b
3	phoX	ac
4	psip1	c

5) DCM_MT

Kruskal Wallis Test

n	statistic	df	p
196	9.397693	3	0.0244

Dunn's Test (Holm's Correction)

Comparison	group1	group2	n1	n2	statistic	p	p.adj	p.adj.signif
phoAaty - phoD	phoAaty	phoD	49	49	-2.73573	0.006224	0.037346	*
phoAaty - phoX	phoAaty	phoX	49	49	-1.73519	0.082706	0.330825	ns
phoAaty - psip1	phoAaty	psip1	49	49	-2.56535	0.010307	0.051535	ns
phoD - phoX	phoD	phoX	49	49	1.000531	0.317053	0.95116	ns
phoD - psip1	phoD	psip1	49	49	0.170372	0.864717	0.95116	ns
phoX - psip1	phoX	psip1	49	49	-0.83016	0.406449	0.95116	ns

	Gene	Group
1	phoAaty	a
2	phoD	b
3	phoX	ab
4	psip1	ab

6) MES_MT

Kruskal Wallis Test

n	statistic	df	p
104	54.91884	3	7.15E-12

Dunn's Test (Holm's Correction)

Comparison	group1	group2	n1	n2	statistic	p	p.adj	p.adj.signif
phoAaty - phoD	phoAaty	phoD	26	26	0.591094	0.554458	1	ns
phoAaty - phoX	phoAaty	phoX	26	26	6.21785	5.04E-10	2.81E-09	****
phoAaty - psip1	phoAaty	psip1	26	26	-0.01137	0.99093	1	ns
phoD - phoX	phoD	phoX	26	26	5.626757	1.84E-08	7.35E-08	****
phoD - psip1	phoD	psip1	26	26	-0.60246	0.546867	1	ns
phoX - psip1	phoX	psip1	26	26	-6.22922	4.69E-10	2.81E-09	****

	Gene	Group
1	phoAaty	a
2	phoD	a
3	phoX	b
4	psip1	a

7) MIX_MT

Kruskal Wallis Test

n	statistic	df	p
28	4.132961	3	0.247

Dunn's Test (Holm's Correction)

Comparison	group1	group2	n1	n2	statistic	p	p.adj	p.adj.signif
phoAaty - phoD	phoAaty	phoD	7	7	-1.0802	0.280052	1	ns
phoAaty - phoX	phoAaty	phoX	7	7	0.056853	0.954662	1	ns
phoAaty - psip1	phoAaty	psip1	7	7	-1.62978	0.103148	0.550043	ns
phoD - phoX	phoD	phoX	7	7	1.137056	0.255515	1	ns
phoD - psip1	phoD	psip1	7	7	-0.54958	0.582609	1	ns
phoX - psip1	phoX	psip1	7	7	-1.68663	0.091674	0.550043	ns

	Gene	Group
1	phoAaty	a
2	phoD	a
3	phoX	a
4	psip1	a

8) SRF_MT

Kruskal Wallis Test

n	statistic	df	p
412	25.99512	3	9.56E-06

Dunn's Test (Holm's Correction)

Comparison	group1	group2	n1	n2	statistic	p	p.adj	p.adj.signif
phoAaty - phoD	phoAaty	phoD	103	103	-5.03632	4.75E-07	2.85E-06	****
phoAaty - phoX	phoAaty	phoX	103	103	-2.83105	0.00464	0.018558	*
phoAaty - psip1	phoAaty	psip1	103	103	-3.19983	0.001375	0.006875	**
phoD - phoX	phoD	phoX	103	103	2.205277	0.027435	0.082304	ns
phoD - psip1	phoD	psip1	103	103	1.836488	0.066285	0.132571	ns
phoX - psip1	phoX	psip1	103	103	-0.36879	0.712285	0.712285	ns

	Gene	Group
1	phoAaty	a
2	phoD	b
3	phoX	b
4	psip1	b

9) phoAaty_MG

Kruskal Wallis Test

n	statistic	df	p
179	11.01735	3	0.0116

Dunn's Test (Holm's Correction)

Comparison	group1	group2	n1	n2	statistic	p	p.adj	p.adj.signif
DCM - MES	DCM	MES	53	38	-2.72357	0.006458	0.03229	*
DCM - MIX	DCM	MIX	53	5	-1.14127	0.253759	0.946888	ns
DCM - SRF	DCM	SRF	53	83	0.062188	0.950413	1	ns
MES - MIX	MES	MIX	38	5	0.094619	0.924617	1	ns
MES - SRF	MES	SRF	38	83	3.011581	0.002599	0.015593	*
MIX - SRF	MIX	SRF	5	83	1.18322	0.236722	0.946888	ns

	Location	Group
1	DCM	a
2	MES	b
3	MIX	ab
4	SRF	a

10) phoX_MG

Kruskal Wallis Test

n	statistic	df	p
179	78.31586	3	7.05E-17

Dunn's Test (Holm's Correction)

Comparison	group1	group2	n1	n2	statistic	p	p.adj	p.adj.signif
DCM - MES	DCM	MES	53	38	7.647778	2.04E-14	1.02E-13	****
DCM - MIX	DCM	MIX	53	5	0.658851	0.509992	1	ns
DCM - SRF	DCM	SRF	53	83	0.01814	0.985527	1	ns
MES - MIX	MES	MIX	38	5	-2.76927	0.005618	0.022473	*
MES - SRF	MES	SRF	38	83	-8.28345	1.20E-16	7.18E-16	****
MIX - SRF	MIX	SRF	5	83	-0.66244	0.507692	1	ns

	Location	Group
1	DCM	a
2	MES	b
3	MIX	a
4	SRF	a

11) phoD_MG

Kruskal Wallis Test

n	statistic	df	p
178	21.10062	3	1.00E-04

Dunn's Test (Holm's Correction)

Comparison	group1	group2	n1	n2	statistic	p	p.adj	p.adj.signif
DCM - MES	DCM	MES	52	38	3.779594	0.000157	0.000785	***
DCM - MIX	DCM	MIX	52	5	-0.69135	0.489348	1	ns
DCM - SRF	DCM	SRF	52	83	-0.11865	0.905553	1	ns
MES - MIX	MES	MIX	38	5	-2.37601	0.017501	0.070004	ns
MES - SRF	MES	SRF	38	83	-4.22537	2.39E-05	0.000143	***
MIX - SRF	MIX	SRF	5	83	0.657388	0.510932	1	ns

	Location	Group
1	DCM	a
2	MES	b
3	MIX	ab
4	SRF	a

12) psip1_MG

Kruskal Wallis Test

n	statistic	df	p
179	10.58875	3	0.0142

Dunn's Test (Holm's Correction)

Comparison	group1	group2	n1	n2	statistic	p	p.adj	p.adj.signif
DCM - MES	DCM	MES	53	38	-2.14615	0.031861	0.159307	ns
DCM - MIX	DCM	MIX	53	5	-1.1763	0.239475	0.718426	ns
DCM - SRF	DCM	SRF	53	83	0.785203	0.432335	0.86467	ns
MES - MIX	MES	MIX	38	5	-0.19784	0.843172	0.86467	ns
MES - SRF	MES	SRF	38	83	3.033977	0.002414	0.014481	*
MIX - SRF	MIX	SRF	5	83	1.494882	0.134945	0.539781	ns

	Location	Group
1	DCM	ab
2	MES	a
3	MIX	ab
4	SRF	b

13) phoAaty_MT

Kruskal Wallis Test

n	statistic	df	p
185	13.8679	3	0.00309

Dunn's Test (Holm's Correction)

Comparison	group1	group2	n1	n2	statistic	p	p.adj	p.adj.signif
DCM - MES	DCM	MES	49	26	-2.88366	0.003931	0.019654	*
DCM - MIX	DCM	MIX	49	7	0.228683	0.819115	1	ns
DCM - SRF	DCM	SRF	49	103	0.642171	0.520762	1	ns
MES - MIX	MES	MIX	26	7	1.860117	0.062869	0.251476	ns
MES - SRF	MES	SRF	26	103	3.695636	0.000219	0.001316	**
MIX - SRF	MIX	SRF	7	103	0.048751	0.961118	1	ns

	Location	Group
1	DCM	a
2	MES	b
3	MIX	ab
4	SRF	a

14) phoX_MT

Kruskal Wallis Test

n	statistic	df	p
185	32.80043	3	3.55E-07

Dunn's Test (Holm's Correction)

Comparison	group1	group2	n1	n2	statistic	p	p.adj	p.adj.signif
DCM - MES	DCM	MES	49	26	5.135476	2.81E-07	1.41E-06	****
DCM - MIX	DCM	MIX	49	7	0.625427	0.531691	1	ns
DCM - SRF	DCM	SRF	49	103	0.273687	0.784325	1	ns
MES - MIX	MES	MIX	26	7	-2.33273	0.019662	0.078648	ns
MES - SRF	MES	SRF	26	103	-5.46083	4.74E-08	2.84E-07	****
MIX - SRF	MIX	SRF	7	103	-0.52539	0.599314	1	ns

	Location	Group
1	DCM	a
2	MES	b
3	MIX	ab
4	SRF	a

15) phoD_MT

Kruskal Wallis Test

n	statistic	df	p
185	0.701944	3	0.873

Dunn's Test (Holm's Correction)

Comparison	group1	group2	n1	n2	statistic	p	p.adj	p.adj.signif
DCM - MES	DCM	MES	49	26	-0.64316	0.520118	1	ns
DCM - MIX	DCM	MIX	49	7	0.151138	0.879866	1	ns
DCM - SRF	DCM	SRF	49	103	-0.63966	0.522392	1	ns
MES - MIX	MES	MIX	26	7	0.509894	0.610126	1	ns
MES - SRF	MES	SRF	26	103	0.205228	0.837394	1	ns
MIX - SRF	MIX	SRF	7	103	-0.44055	0.659538	1	ns

	Location	Group
1	DCM	a
2	MES	a
3	MIX	a
4	SRF	a

16) psip1_MT

Kruskal Wallis Test

n	statistic	df	p
185	5.483579	3	0.14

Dunn's Test (Holm's Correction)

Comparison	group1	group2	n1	n2	statistic	p	p.adj	p.adj.signif
DCM - MES	DCM	MES	49	26	-1.45006	0.147043	0.735215	ns
DCM - MIX	DCM	MIX	49	7	-0.63707	0.524076	1	ns
DCM - SRF	DCM	SRF	49	103	0.771177	0.440602	1	ns
MES - MIX	MES	MIX	26	7	0.221718	0.824533	1	ns
MES - SRF	MES	SRF	26	103	2.212802	0.026911	0.161468	ns
MIX - SRF	MIX	SRF	7	103	1.00167	0.316503	1	ns

	Location	Group
1	DCM	a
2	MES	a
3	MIX	a
4	SRF	a

Table S3. Statistical comparisons of APase abundance across oceanic regions in the TARA Oceans dataset. Within each dataset, Kruskal Wallis test results followed by Holm's corrected post hoc Dunn's test for multiple comparisons are presented. These test results are also presented in an alternate format; each category is assigned a group by letter or letters. Categories sharing the same letter are not statistically different from one another. Abbreviations MG- metagenome; MT- metatranscriptomes; DCM- deep chlorophyll maxima; SRF- surface; MES- mesopelagic; MIX; wind mixed layer; AO- Arctic Ocean; IO- Indian Ocean; MS- Mediterranean Sea; NAO-North Atlantic Ocean; NPO- North Pacific Ocean; RS- Red Sea; SAO- South Atlantic Ocean; SO- Southern Ocean; SPO- South Pacific Ocean. Sheets are labelled to reflect the test being conducted; thus AO_MG contains test data for comparisons between abundance of the four phosphatases within the Arctic Ocean samples of the TARA metagenome (MG).

1) AO_MG

Kruskal Wallis Test

n	statistic	df	p
112	36.13953	3	7.00E-08

Dunn's Test (Holm's Correction)

Comparison	group1	group2	n1	n2	statistic	p	p.adj	p.adj.signif
phoAaty - phoD	phoAaty	phoD	28	28	0	1	1	ns
phoAaty - phoX	phoAaty	phoX	28	28	4.908464	9.18E-07	5.51E-06	****
phoAaty - psip1	phoAaty	psip1	28	28	0	1	1	ns
phoD - phoX	phoD	phoX	28	28	4.908464	9.18E-07	5.51E-06	****
phoD - psip1	phoD	psip1	28	28	0	1	1	ns
phoX - psip1	phoX	psip1	28	28	-4.90846	9.18E-07	5.51E-06	****

	Gene	Group
1	phoAaty	a
2	phoD	a
3	phoX	b
4	psip1	a

2) IO_MG

Kruskal Wallis Test

n	statistic	df	p
84	38.93657	3	1.79E-08

Dunn's Test (Holm's Correction)

Comparison	group1	group2	n1	n2	statistic	p	p.adj	p.adj.signif
phoAaty - phoD	phoAaty	phoD	21	21	-6.17386	6.66E-10	4.00E-09	****
phoAaty - phoX	phoAaty	phoX	21	21	-2.31479	0.020624	0.041249	*
phoAaty - psip1	phoAaty	psip1	21	21	-2.95905	0.003086	0.009258	**
phoD - phoX	phoD	phoX	21	21	3.859064	0.000114	0.000569	***
phoD - psip1	phoD	psip1	21	21	3.214808	0.001305	0.005221	**
phoX - psip1	phoX	psip1	21	21	-0.64426	0.519409	0.519409	ns

	Gene	Group
1	phoAaty	a
2	phoD	b
3	phoX	c
4	psip1	c

3) MS_MG

Kruskal Wallis Test

n	statistic	df	p
48	38.61127	3	2.10E-08

Dunn's Test (Holm's Correction)

Comparison	group1	group2	n1	n2	statistic	p	p.adj	p.adj.signif
phoAaty - phoD	phoAaty	phoD	12	12	-4.97242	6.61E-07	3.31E-06	****
phoAaty - phoX	phoAaty	phoX	12	12	-5.23489	1.65E-07	9.90E-07	****
phoAaty - psip1	phoAaty	psip1	12	12	-1.80815	0.070583	0.141165	ns
phoD - phoX	phoD	phoX	12	12	-0.26247	0.792956	0.792956	ns
phoD - psip1	phoD	psip1	12	12	3.164268	0.001555	0.004664	**
phoX - psip1	phoX	psip1	12	12	3.426742	0.000611	0.002443	**

	Gene	Group
1	phoAaty	a
2	phoD	b
3	phoX	b
4	psip1	a

4) NAO_MG

Kruskal Wallis Test

n	statistic	df	p
63	21.09816	3	1.00E-04

Dunn's Test (Holm's Correction)

Comparison	group1	group2	n1	n2	statistic	p	p.adj	p.adj.signif
phoAaty - phoD	phoAaty	phoD	16	15	-4.43249	9.32E-06	5.59E-05	****
phoAaty - phoX	phoAaty	phoX	16	16	-3.02164	0.002514	0.011237	*
phoAaty - psip1	phoAaty	psip1	16	16	-3.05543	0.002247	0.011237	*
phoD - phoX	phoD	phoX	15	16	1.459979	0.144296	0.432887	ns
phoD - psip1	phoD	psip1	15	16	1.42674	0.153655	0.432887	ns
phoX - psip1	phoX	psip1	16	16	-0.03379	0.973046	0.973046	ns

	Gene	Group
1	phoAaty	a
2	phoD	b
3	phoX	b
4	psip1	b

5) NPO_MG

Kruskal Wallis Test

n	statistic	df	p
44	8.750631	3	0.0328

Dunn's Test (Holm's Correction)

Comparison	group1	group2	n1	n2	statistic	p	p.adj	p.adj.signif
phoAaty - phoD	phoAaty	phoD	11	11	-2.07932	0.037588	0.164583	ns
phoAaty - phoX	phoAaty	phoX	11	11	-2.1331	0.032917	0.164583	ns
phoAaty - psip1	phoAaty	psip1	11	11	-2.77841	0.005463	0.032776	*
phoD - phoX	phoD	phoX	11	11	-0.05378	0.957114	1	ns
phoD - psip1	phoD	psip1	11	11	-0.69908	0.4845	1	ns
phoX - psip1	phoX	psip1	11	11	-0.64531	0.518728	1	ns

	Gene	Group
1	phoAaty	a
2	phoD	ab
3	phoX	ab
4	psip1	b

6) RS_MG

Kruskal Wallis Test

n	statistic	df	p
24	21.35089	3	8.90E-05

Dunn's Test (Holm's Correction)

Comparison	group1	group2	n1	n2	statistic	p	p.adj	p.adj.signif
phoAaty - phoD	phoAaty	phoD	6	6	-4.34828	1.37E-05	8.23E-05	****
phoAaty - phoX	phoAaty	phoX	6	6	-2.8715	0.004085	0.016341	*
phoAaty - psip1	phoAaty	psip1	6	6	-1.31269	0.189288	0.357119	ns
phoD - phoX	phoD	phoX	6	6	1.476774	0.139736	0.357119	ns
phoD - psip1	phoD	psip1	6	6	3.03559	0.002401	0.012003	*
phoX - psip1	phoX	psip1	6	6	1.558817	0.11904	0.357119	ns

	Gene	Group
1	phoAaty	a
2	phoD	b
3	phoX	bc
4	psip1	ac

7) SAO_MG

Kruskal Wallis Test

n	statistic	df	p
56	18.21775	3	0.000397

Dunn's Test (Holm's Correction)

Comparison	group1	group2	n1	n2	statistic	p	p.adj	p.adj.signif
phoAaty - phoD	phoAaty	phoD	14	14	-4.1675	3.08E-05	0.000185	***
phoAaty - phoX	phoAaty	phoX	14	14	-1.33312	0.182493	0.364986	ns
phoAaty - psip1	phoAaty	psip1	14	14	-2.08975	0.03664	0.14656	ns
phoD - phoX	phoD	phoX	14	14	2.834377	0.004592	0.022958	*
phoD - psip1	phoD	psip1	14	14	2.077743	0.037733	0.14656	ns
phoX - psip1	phoX	psip1	14	14	-0.75663	0.449269	0.449269	ns

	Gene	Group
1	phoAaty	a
2	phoD	b
3	phoX	a
4	psip1	ab

8) SPO_MG

Kruskal Wallis Test

n	statistic	df	p
100	22.18299	3	5.98E-05

Dunn's Test (Holm's Correction)

Comparison	group1	group2	n1	n2	statistic	p	p.adj	p.adj.signif
phoAaty - phoD	phoAaty	phoD	25	25	-3.93642	8.27E-05	0.000496	***
phoAaty - phoX	phoAaty	phoX	25	25	-3.84666	0.00012	0.000599	***
phoAaty - psip1	phoAaty	psip1	25	25	-3.74409	0.000181	0.000724	***
phoD - phoX	phoD	phoX	25	25	0.089755	0.928482	1	ns
phoD - psip1	phoD	psip1	25	25	0.192333	0.847481	1	ns
phoX - psip1	phoX	psip1	25	25	0.102578	0.918298	1	ns

	Gene	Group
1	phoAaty	a
2	phoD	b
3	phoX	b
4	psip1	b

9) AO_MT

Kruskal Wallis Test

n	statistic	df	p
108	29.41185	3	1.83E-06

Dunn's Test (Holm's Correction)

Comparison	group1	group2	n1	n2	statistic	p	p.adj	p.adj.signif
phoAaty - phoD	phoAaty	phoD	27	27	-4.68893	2.75E-06	1.65E-05	****
phoAaty - phoX	phoAaty	phoX	27	27	-2.8716	0.004084	0.016336	*
phoAaty - psip1	phoAaty	psip1	27	27	-4.68893	2.75E-06	1.65E-05	****
phoD - phoX	phoD	phoX	27	27	1.817339	0.069165	0.207496	ns
phoD - psip1	phoD	psip1	27	27	0	1	1	ns
phoX - psip1	phoX	psip1	27	27	-1.81734	0.069165	0.207496	ns

	Gene	Group
1	phoAaty	a
2	phoD	b
3	phoX	b
4	psip1	b

10) IO_MT

Kruskal Wallis Test

n	statistic	df	p
72	24.79745	3	1.70E-05

Dunn's Test (Holm's Correction)

Comparison	group1	group2	n1	n2	statistic	p	p.adj	p.adj.signif
phoAaty - phoD	phoAaty	phoD	18	18	-4.25703	2.07E-05	0.000124	***
phoAaty - phoX	phoAaty	phoX	18	18	-3.58948	0.000331	0.001325	**
phoAaty - psip1	phoAaty	psip1	18	18	-4.21326	2.52E-05	0.000126	***
phoD - phoX	phoD	phoX	18	18	0.667555	0.504417	1	ns
phoD - psip1	phoD	psip1	18	18	0.043774	0.965084	1	ns
phoX - psip1	phoX	psip1	18	18	-0.62378	0.532771	1	ns

	Gene	Group
1	phoAaty	a
2	phoD	b
3	phoX	b
4	psip1	b

11) MS_MT

Kruskal Wallis Test

n	statistic	df	p
28	21.65601	3	7.69E-05

Dunn's Test (Holm's Correction)

Comparison	group1	group2	n1	n2	statistic	p	p.adj	p.adj.signif
phoAaty - phoD	phoAaty	phoD	7	7	-3.94969	7.83E-05	0.00047	***
phoAaty - phoX	phoAaty	phoX	7	7	-3.49458	0.000475	0.002374	**
phoAaty - psip1	phoAaty	psip1	7	7	-1.07275	0.283381	0.566763	ns
phoD - phoX	phoD	phoX	7	7	0.455108	0.649032	0.649032	ns
phoD - psip1	phoD	psip1	7	7	2.876933	0.004016	0.016062	*
phoX - psip1	phoX	psip1	7	7	2.421825	0.015443	0.046328	*

	Gene	Group
1	phoAaty	a
2	phoD	b
3	phoX	b
4	psip1	a

12) NAO_MT

Kruskal Wallis Test

n	statistic	df	p
68	15.96579	3	0.00115

Dunn's Test (Holm's Correction)

Comparison	group1	group2	n1	n2	statistic	p	p.adj	p.adj.signif
phoAaty - phoD	phoAaty	phoD	17	17	-3.91982	8.86E-05	0.000532	***
phoAaty - phoX	phoAaty	phoX	17	17	-2.31191	0.020782	0.086224	ns
phoAaty - psip1	phoAaty	psip1	17	17	-1.53838	0.123956	0.323565	ns
phoD - phoX	phoD	phoX	17	17	1.60791	0.107855	0.323565	ns
phoD - psip1	phoD	psip1	17	17	2.381445	0.017245	0.086224	ns
phoX - psip1	phoX	psip1	17	17	0.773535	0.439206	0.439206	ns

	Gene	Group
1	phoAaty	a
2	phoD	b
3	phoX	ab
4	psip1	ab

13) NPO_MT

Kruskal Wallis Test

n	statistic	df	p
80	0.546976	3	0.908

Dunn's Test (Holm's Correction)

Comparison	group1	group2	n1	n2	statistic	p	p.adj	p.adj.signif
phoAaty - phoD	phoAaty	phoD	20	20	-0.54917	0.582886	1	ns
phoAaty - phoX	phoAaty	phoX	20	20	-0.62269	0.533491	1	ns
phoAaty - psip1	phoAaty	psip1	20	20	-0.62701	0.530653	1	ns
phoD - phoX	phoD	phoX	20	20	-0.07351	0.941399	1	ns
phoD - psip1	phoD	psip1	20	20	-0.07784	0.937959	1	ns
phoX - psip1	phoX	psip1	20	20	-0.00432	0.99655	1	ns

	Gene	Group
1	phoAaty	a
2	phoD	a
3	phoX	a
4	psip1	a

14) RS_MT

Kruskal Wallis Test

n	statistic	df	p
12	7.001208	3	0.0719

Dunn's Test (Holm's Correction)

Comparison	group1	group2	n1	n2	statistic	p	p.adj	p.adj.signif
phoAaty - phoD	phoAaty	phoD	3	3	-2.53573	0.011221	0.067327	ns
phoAaty - phoX	phoAaty	phoX	3	3	-1.9018	0.057197	0.285986	ns
phoAaty - psip1	phoAaty	psip1	3	3	-1.3255	0.185006	0.740024	ns
phoD - phoX	phoD	phoX	3	3	0.633934	0.526124	1	ns
phoD - psip1	phoD	psip1	3	3	1.210237	0.226188	0.740024	ns
phoX - psip1	phoX	psip1	3	3	0.576303	0.56441	1	ns

	Gene	Group
1	phoAaty	a
2	phoD	a
3	phoX	a
4	psip1	a

15) SAO_MT

Kruskal Wallis Test

n	statistic	df	p
68	13.38028	3	0.00388

Dunn's Test (Holm's Correction)

Comparison	group1	group2	n1	n2	statistic	p	p.adj	p.adj.signif
phoAaty - phoD	phoAaty	phoD	17	17	-3.63112	0.000282	0.001693	**
phoAaty - phoX	phoAaty	phoX	17	17	-2.04155	0.041196	0.20598	ns
phoAaty - psip1	phoAaty	psip1	17	17	-1.59972	0.10966	0.32898	ns
phoD - phoX	phoD	phoX	17	17	1.589566	0.111933	0.32898	ns
phoD - psip1	phoD	psip1	17	17	2.031394	0.042215	0.20598	ns
phoX - psip1	phoX	psip1	17	17	0.441828	0.658614	0.658614	ns

	Gene	Group
1	phoAaty	a
2	phoD	b
3	phoX	ab
4	psip1	ab

16) SO_MT

Kruskal Wallis Test

n	statistic	df	p
32	3.908309	3	0.272

Dunn's Test (Holm's Correction)

Comparison	group1	group2	n1	n2	statistic	p	p.adj	p.adj.signif
phoAaty - phoD	phoAaty	phoD	8	8	-0.84297	0.399244	1	ns
phoAaty - phoX	phoAaty	phoX	8	8	0.842972	0.399244	1	ns
phoAaty - psip1	phoAaty	psip1	8	8	-0.84297	0.399244	1	ns
phoD - phoX	phoD	phoX	8	8	1.685944	0.091807	0.55084	ns
phoD - psip1	phoD	psip1	8	8	0	1	1	ns
phoX - psip1	phoX	psip1	8	8	-1.68594	0.091807	0.55084	ns

	Gene	Group
1	phoAaty	a
2	phoD	a
3	phoX	a
4	psip1	a

17) SPO_MT

Kruskal Wallis Test

n	statistic	df	p
140	3.217939	3	0.359

Dunn's Test (Holm's Correction)

Comparison	group1	group2	n1	n2	statistic	p	p.adj	p.adj.signif
phoAaty - phoD	phoAaty	phoD	35	35	-1.71808	0.085782	0.514691	ns
phoAaty - phoX	phoAaty	phoX	35	35	-1.30091	0.193289	0.966445	ns
phoAaty - psip1	phoAaty	psip1	35	35	-0.94412	0.345108	1	ns
phoD - phoX	phoD	phoX	35	35	0.41717	0.676554	1	ns
phoD - psip1	phoD	psip1	35	35	0.77396	0.438955	1	ns
phoX - psip1	phoX	psip1	35	35	0.35679	0.721249	1	ns

	Gene	Group
1	phoAaty	a
2	phoD	a
3	phoX	a
4	psip1	a

Table S4. Individual correlations (Correlations tab) and linear regression models (Linear Regression Models tab) of TARA environmental variables and *psip1* abundance in metagenomes and metatranscriptomes.

			TARA Metagenomics				TARA Metatranscriptomics			
Independent variable	Units	Log transformed	rho	p	adj_p	Significant correlation	rho	p	adj_p	Significant correlation
Iron at 5m	mmol L^-1	1	5.71E-01	3.03E-07	8.47E-06	1	5.89E-01	3.31E-05	4.64E-04	1
'PO4 moll'	mol L^-1	0	-6.39E-01	1.76E-06	2.47E-05	1	-5.58E-01	8.98E-04	8.38E-03	1
'Ammonium_5mmoll'	mmol L^-1	1	-4.95E-01	1.55E-05	0.000145	1	-4.71E-01	1.45E-03	0.010122	1
'Salinity PSU'	PSU	1	0.47	0.00	0.00	1	0.42	0.01	0.02	1
Distance_coast	km	0	-3.54E-01	0.002872	0.013681	1	-4.63E-01	0.002019	0.010999	1
'PIC mol m3'	mol m^-3	0	-0.3814	0.00342	0.013681	1	-0.49107	0.002357	0.010999	1
'NO3_NO2moll'	mol L^-1	0	-0.43022	0.003181	0.013681	1	-0.32285	0.071504	0.222457	0
Density	kg m^-3	1	3.38E-01	0.004857	0.016999	1	2.61E-01	0.094514	0.264639	0
Nitrate	mol L^-1	0	-0.42594	0.009593	0.029845	1	-0.02882	0.89365	0.926748	0
'Si moll'	mol L^-1	0	-0.33505	0.013266	0.037145	1	-0.12249	0.463785	0.64861	0
'CDOM ppb QSE'	ppb	1	-0.48067	0.027407	0.069762	0	-0.43826	0.460406	0.64861	0
'NO2 moll'	mol L^-1	0	-0.30027	0.030556	0.071297	0	-0.19594	0.274472	0.548943	0
'Alkalinity mol kg'	mol kg^-1	0	-3.84E-01	0.048106	0.096212	0	-5.30E-01	0.016316	0.057107	0
'HCO3 mol kg'	mol kg^-1	1	0.821198	0.045097	0.096212	0	0	0	0	1
'MLD m'	m	1	-0.23794	0.052507	0.098014	0	0.089919	0.571188	0.695359	0
'Depth m'	m	1	-0.22546	0.064516	0.112903	0	-0.098	0.531823	0.676866	0
'O2 mol kg'	mol kg^-1	1	0.224278	0.0797	0.131271	0	0.135628	0.391772	0.64861	0
'Chlorophyll_a mg m3'	mg m^-3	0	-0.22181	0.110426	0.171774	0	-0.05668	0.735371	0.823615	0
'Nitrate_5mmoll'	mmol L^-1	0	-0.18417	0.129807	0.191294	0	-0.1968	0.205905	0.480446	0
'POC g kg'	g kg^-1	1	-1.53E-01	0.242699	0.339779	0	-1.71E-01	0.312188	0.582751	0
'CO2 mol kg'	mol kg^-1	1	-0.25408	0.266399	0.355199	0	0.095251	0.716125	0.823615	0
'pH'		1	0.22258	0.332161	0.42275	0	-0.18202	0.484426	0.64861	0

'NPP_C mg m2 day'	mg m^-2 d^-1	1	-0.08452	0.499863	0.608529	0	-0.17268	0.398904	0.64861	0
'PAR mol Quanta m2 day'	mol Q m^-2 d^-1	0	0.030721	0.823805	0.887174	0	-0.01361	0.937216	0.937216	0
'Carbon Total mol kg'	mol kg^-1	0	0.043308	0.793489	0.887174	0	0.216559	0.268348	0.548943	0
'CO3 mol kg'	mol kg^-1	0	0.046182	0.777202	0.887174	0	0.281151	0.147253	0.374825	0
'Temperature C'	degC	1	0.01	0.93	0.93	0	0.11	0.49	0.65	0
'Nitrite_5mmoll'	mmol L^-1	0	0.013055	0.915214	0.928156	0	0.03997	0.799123	0.860594	0

TARA Metagenomics

**Variable**	**Coefficient**	**Standard Error**	**t Statistic**	**p Value**
'PO4moll'	-0.16887	0.022165	-7.61883	9.49E-10
'NO3moll'	0.113332	0.037875	2.992292	0.004401
'Simoll'	0.041687	0.02136	1.951602	0.056959
'Densitykgm3'	-0.02685	0.016023	-1.6757	0.100439
'NO3_NO2moll'	-0.02432	0.026296	-0.92497	0.359708
'(Intercept)'	0.321124	0.569033	0.564333	5.75E-01
'Ammonium_5mmoll'	0.501919	0.92265	0.543997	0.589015
'Iron_5mmoll'	-18.6725	50.89156	-0.36691	0.715334
'PICmolm3'	-0.0083	0.036842	-0.22534	0.822695

Model Fit:

Number of observations: 56, Error degrees of freedom: 47

Root Mean Squared Error: 0.134

R-squared: 0.692, Adjusted R-Squared: 0.64

F-statistic vs. constant model: 13.2, p-value = 9.02e-10

TARA Metatranscriptomics

Variable	Coefficient	Standard Error	t Statistic	p Value
'(Intercept)'	52.76474	33.20585	1.589019	0.119383
'Densitykgm3'	-1.78505	0.991882	-1.79966	0.078933
'NO3moll'	4.860062	2.257623	2.152734	0.036994
'Iron_5mmoll'	1085.584	3980.141	0.27275	0.786351
'Ammonium_5mmoll'	9.636988	25.12598	0.383547	0.703204
'PICmolm3'	1.818796	1.961027	0.927471	0.358859
'PO4moll'	-3.5874	1.309929	-2.73862	0.008942
'NO3_NO2moll'	-3.17892	1.895745	-1.67687	0.100824
'Simoll'	1.996227	1.288839	1.548857	0.128746

Model Fit:

Number of observations: 52, Error degrees of freedom: 43

Root Mean Squared Error: 7.3

R-squared: 0.432, Adjusted R-Squared: 0.326

F-statistic vs. constant model: 4.09, p-value = 0.00109

Table S5. Transcripts per Million (TPM) of phosphatases from all stations along the AMT22 and AMT23 transects.

STATION	LATITUDE	LONGITUDE	Phosphate concentration [μM]	*TRANSCRIPTS PER MILLION (TPM)*			
				psip1	*phoX*	*phoD*	*phoAaty*
**AMT22_18_18_CT**	28.94	-34.67	0.02	1.39E+02	8.997E-02	3.33E-02	7.87E+01
**AMT22_3_4_CT**	48.93	-16.37	0.15	1.10E-01	0	0	5.68E-01
**AMT22_53_53_CT**	-21.25	-24.85	0.16	1.81E-01	0	0	6.19E-01
**AMT22_CTD4**	47.88	-17.27	0.06	3.29E-01	3.336E-01	0	2.13E-01
**AMT22_CTD75**	-45.50	-51.32	0.62	3.77E-01	0	0	3.18E-02
**AMT22_75_75_CT**	-47.50	-53.32	0.62	1.21E-01	0	0	3.50E-01
**AMT22_75_75_SS**	-43.50	-49.32	0.62	7.46E-02	0	0	4.94E-02
**AMT23_11_15_CT**	30.369	-23.157	0.02	7.83E+00	0	0	4.90E+01
**AMT23_11_15_DS**	23.079	-26.815	0.02	9.15E-01	0	0	9.19E+00
**AMT23_37_46_CT**	-22.571	-25.911	0.08	1.80E-01	0	0	1.79E-01
**AMT23_37_46_DS**	-35.159	-38.274	0.08	1.27E-01	5.64E-02	5.45E-03	1.80E-01
**AMT23_39_49_DS**	-23.162	-28.339	0.13	1.93E-01	0	0	2.00E-01
**AMT23_3_5_CT**	44.112	-14.255	0.02	1.55E-01	0	1.34E-02	7.44E-01
**AMT23_3_5_DS**	42.112	-12.255	0.02	7.69E-02	0	0	5.54E-02
**AMT23_54_65_CT**	-46.273	-50.744	0.38	1.72E-01	0	0	2.92E-01
**AMT23_54_65_DS**	-42.273	-46.744	0.38	1.20E-01	0	0	1.12E-02

Paired-end reads of the transcriptomics data obtained from AMT cruises JC079 & JC300 have been deposited in the European Nucleotide Archive (ENA) at EMBL-EBI under accession number PRJEB61548. AMT phosphate data was retrieved from doi.org/10.5285/C73B01F0-C017-4312-E053-6C86ABC08369 and doi:10.5285/215a1e9b-428c-52f5-e053-6c86abc06d17.

Table S6. Statistical comparison of TARA assigned taxonomy of eukaryotic *psip1* transcripts by size fraction. X^2 test results, together with Holm's corrected pairwise comparisons between size fractions are included. Abbreviations: X0.8: >0.8 µm, X0.8_5: 0.8-5 µm, X5_20: 5-20 µm, X20_180: 20-180 µm, X180_2000: 180-2000 µm.

% Abundance by taxa	0.8	0.8_5	05_20	20_180	180_2000
Unknown	45.32877	46.35099	3.785182	5.867096	10.72177
Dinoflagellates	13.91452	12.86621	25.68594	12.90306	39.03505
Haptophytes	33.72217	28.72947	32.26819	5.718626	22.59846
Diatoms	6.160679	7.564613	37.0887	75.3725	26.828
Green Algae	0.873871	4.488724	1.171995	0.138718	0.816726

Chi-square test	n	statistic	p	df	p.signif
1	500	241.2972	3.14E-42	16	****

Pairwise comparisons	n	group1	group2	statistic	p	df	p.adj	p.adj.signif
1	200	X0.8	X0.8_5	3.031902	5.53E-01	4	5.53E-01	ns
2	200	X0.8	X05_20	60.8316	1.94E-12	4	1.36E-11	****
3	200	X0.8	X20_180	109.6243	8.75E-23	4	8.75E-22	****
4	200	X0.8	X180_2000	48.43197	7.67E-10	4	3.84E-09	****
5	200	X0.8_5	X05_20	62.07098	1.06E-12	4	8.48E-12	****
6	200	X0.8_5	X20_180	106.2852	4.51E-22	4	4.06E-21	****
7	200	X0.8_5	X180_2000	49.50022	4.59E-10	4	2.75E-09	****
8	200	X05_20	X20_180	37.08642	1.73E-07	4	5.19E-07	****
9	200	X05_20	X180_2000	9.484946	5.01E-02	4	1.00E-01	ns
10	200	X20_180	X180_2000	48.17017	8.7E-10	4	3.84E-09	****

Supplementary Figures

Figure S1. SDS-PAGE showing the Psip1 purification process. Lanes 1 & 2: Individual elution fractions following purification using a His-TRAP column. Lanes 3, 4, 5, and 6: Individual elution fractions following purification after removing the His-tag. F: Flow through of unbound proteins. L: Molecular weight ladder. The red arrow indicates the Psip1 protein which is approximately 80 kDa in size.

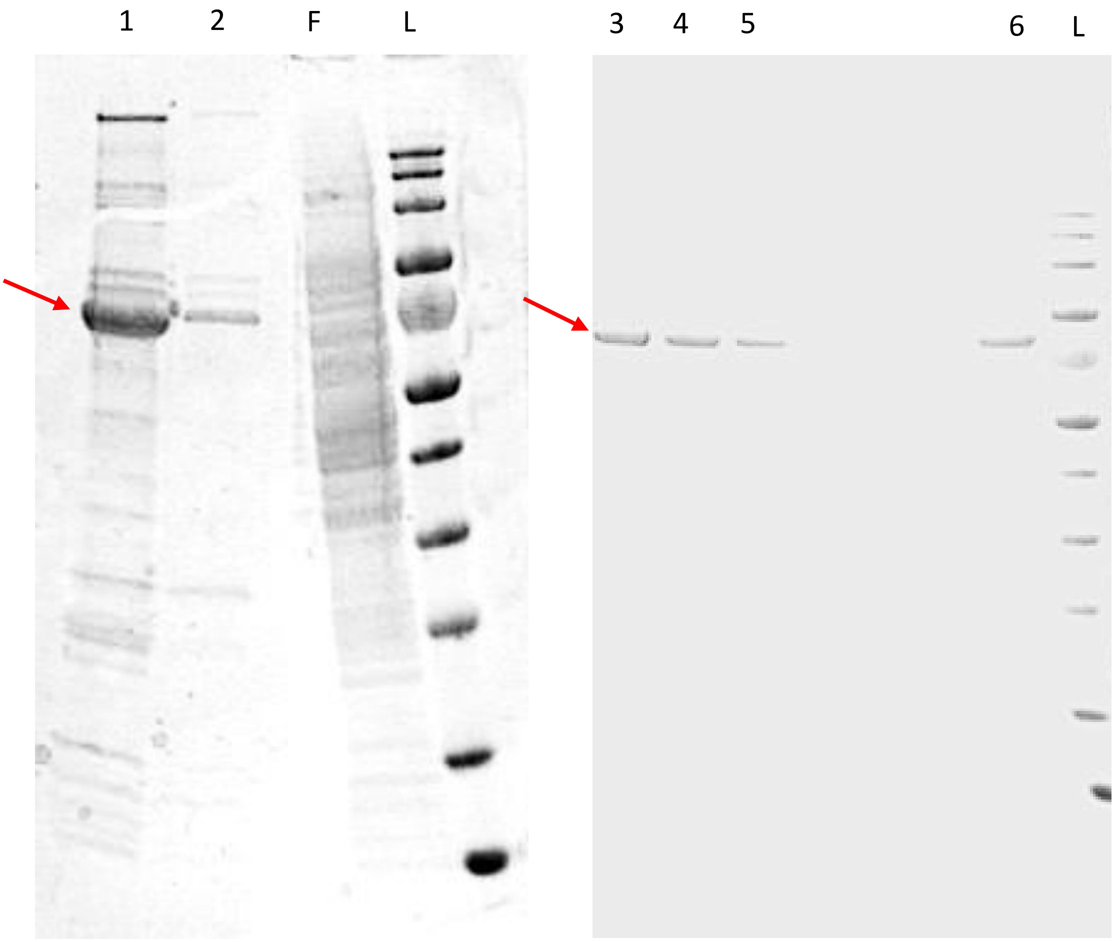

Figure S2. Psip1 phosphatase activity in the presence of specific metal ions (i.e. Ca^{2+}, Mg^{2+}, Mn^{2+}, Co^{2+}, or Fe^{3+}) alone or in combination with calcium. All metals were used at 10 mM concentration except iron (10 µM). Release of *p*-NP from *p*-NPP was measured at 405 nm.

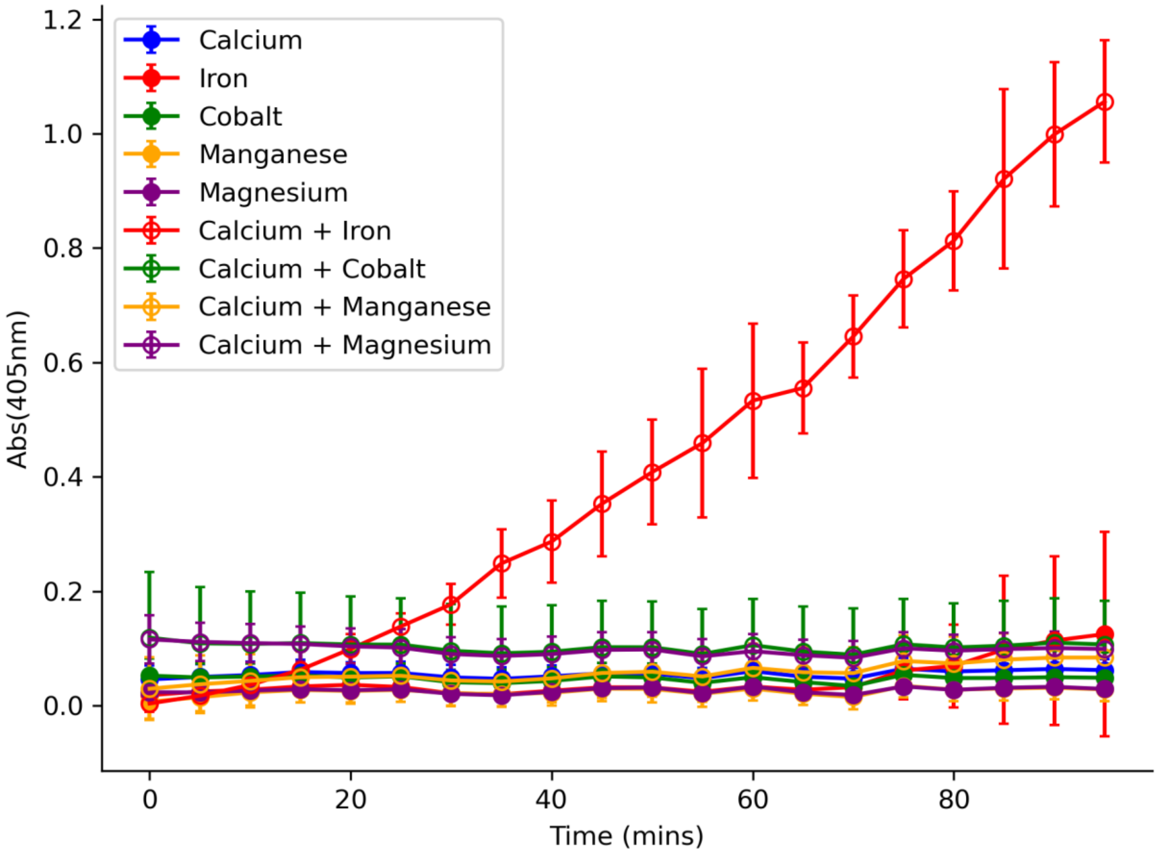

Figure S3. Biochemical characterization of Psip1 from *Prochlorococcus* sp. MED4. A) Michaelis-Menten kinetics of Psip1 activity for *p*NPP was carried by fitting the Michaelis-Menten equation to the V values (nmoles min^{-1} mg $protein^{-1}$) using Scipy 1.10.1 (n=3). (B) Psip1 possesses no phosphodiesterase activity using Bis-*p*NPP as a substrate (n=3). Note the lack of change in absorbance at 405 nm compared to the *Flavobacterium johnsoniae* cell lysate positive control.

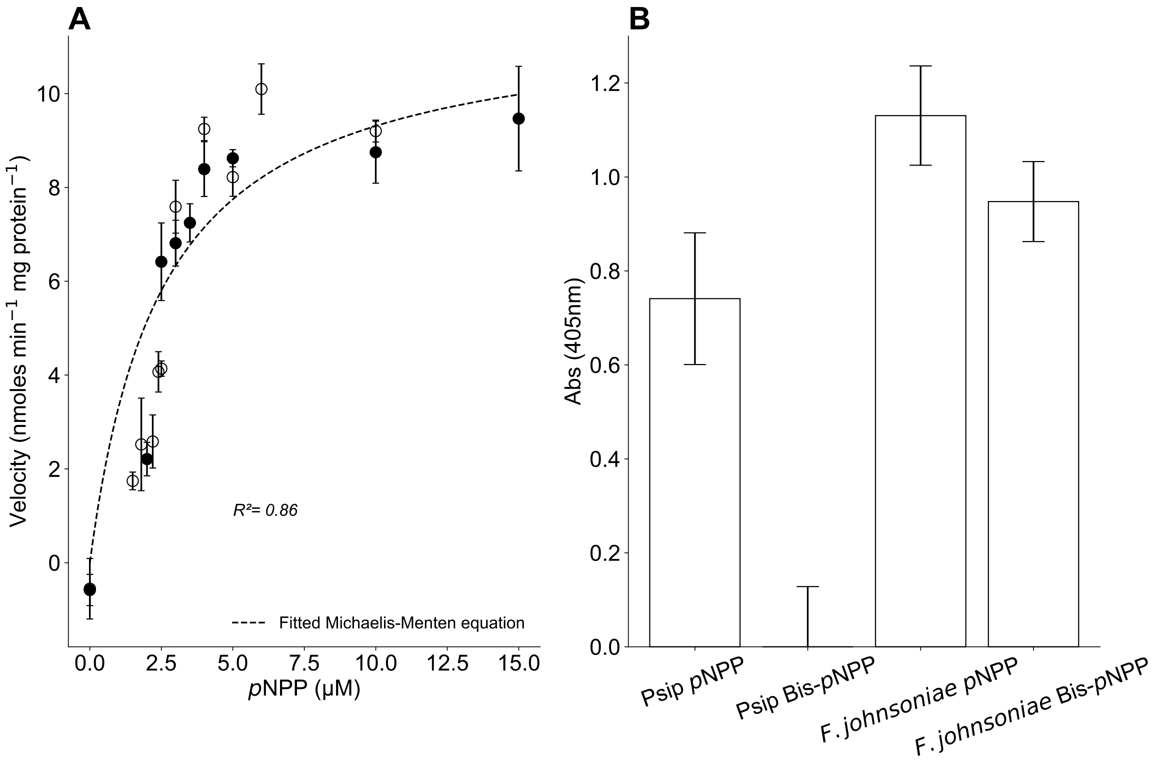

Michaelis-Menten constants (V_{max} and K_m) were obtained using different concentrations of *p*NPP substrate. The reaction buffer comprised 0.1 mM iron (III), 10 mM Ca^{2+}, 20 mM Tris-HCl pH 8.8. The *p*NP calibration curve used 0.5, 1, 2, 5, 10, 20, 30 nmoles standards. Substrate concentrations used for determining kinetic parameters were 0, 0.5, 1, 1.25, 1.5, 1.6, 1.8, 2, 2.2, 2.4, 2.5, 3, 3.5, 4, 4.5, 5, 6, 10 and 15 µM in 20 mM Tris-HCl (pH 8.8) reaction buffer containing iron (0.1 mM) and calcium (10 mM). Controls followed the same procedure, and all conditions and controls were repeated in triplicate. Absorbance was corrected using the standard curve to estimate nmoles per minute, and the rate of reaction normalized to the amount of protein used. Rates were measured every two minutes and curves fitted to the linear part of the reaction. Reaction rates (nmoles/min/mg protein) were plotted against *p*NPP substrate concentration. After plotting, the Michaelis-Menten curve was fitted using the equation V_{max} * ([S]/ (Km + [S])). K_m and V_{max} values were obtained using Python package Scipy 1.10.1 (16).

Figure S4. Michaelis Menten kinetics of Psip1 using the MUF-P substrate in the presence and absence of the following organic P compounds (A) 2 μM glycerol-3-phosphate (G3P), 5 μM phosphoethanolamine (PE), (B) 5 μM phosphocholine (PC), 2.5 μM adenosine monophosphate (AMP), (C) 2 μM glucosamine-6-phosphate (Ga6P), 2 μM glycerol-1-phosphate (G1P). While coefficients of inhibition (K_i) (see Table 1, main text) were calculated by simultaneous non-linear regression using the Michaelis Menten equation for competitive inhibition, the curves presented here were calculated by non-linear regression on each experimental variable using the standard Michaelis Menten equation.

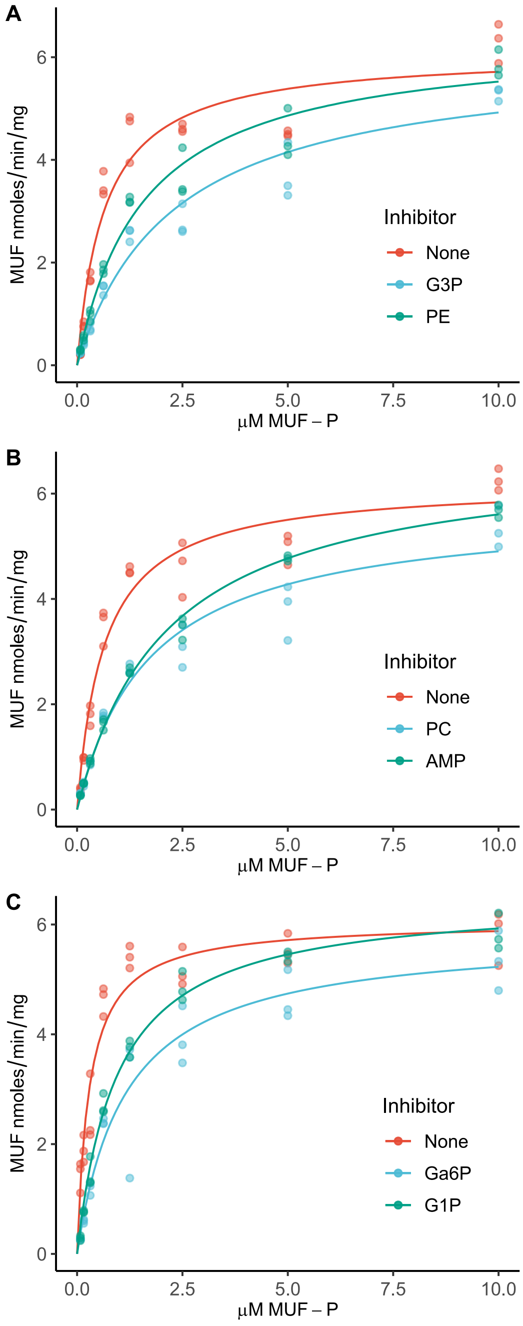

Figure S5. Psip1 induced Pi release from organic P molecules. Pi was detected using ammonium molybdate, and concentrations determined by reference to a standard curve. Data represents mean values of three replicates, and error bars show standard deviation. Equivalent values from minus enzyme controls were subtracted prior to this analysis. All compounds were tested at 100 µM concentration. G3P, glycerol-3-phosphate, G1P, glycerol-1-phosphate, PE, phosphoethanolamine, PC phosphocholine, AMP, adenosine monophosphate, Ga6P, glucosamine-6-phosphate, MUF-P, methylumbelliferyl phosphate, *p*NPP, *para*-nitrophenyl phosphate, Bis-MUF-P, bis-methylumbelliferyl phosphate, Bis-*p*NPP, bis-*para*-nitrophenyl phosphate, GPC, glycerophosphorylcholine, MPn, methylphosphonate, 2AEP, 2-aminoethylphosphonate. All conditions were run for the same length of time, with the same concentration of protein. Pi release was detected for all monoesters tested (G3P, PE, PC, AMP, Ga6P, MUF-P, PNPP). Pi release was detected from one tested diester (GPC), but not Bis-MUF-P or Bis-PNPP (despite detectable MUF release from Bis-MUF-P). No Pi release was detected from phosphonate (direct C-P bond) compounds (MPn, 2AEP).

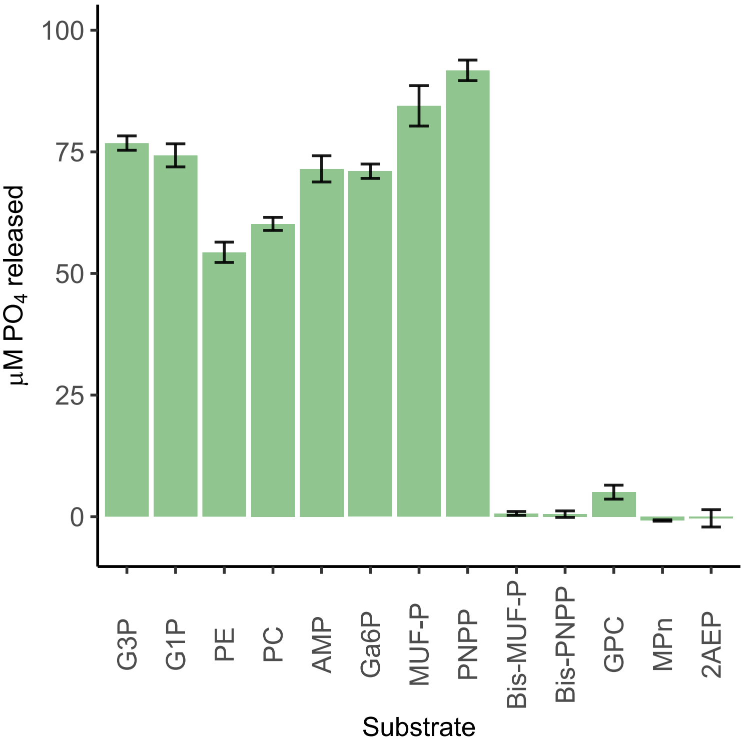

Figure S6. A) Predicted protein structure generated by Alphafold v.2, highlighting the ß -Propeller domain, common in phosphatases and the signal peptide predicted by SignalP5.0. B) SignalP5.0 output file predicting the presence of a signal peptide in *Prochlorococcus* sp. MED4 Psip1, suggesting Psip1 is exported via the Sec pathway.

A

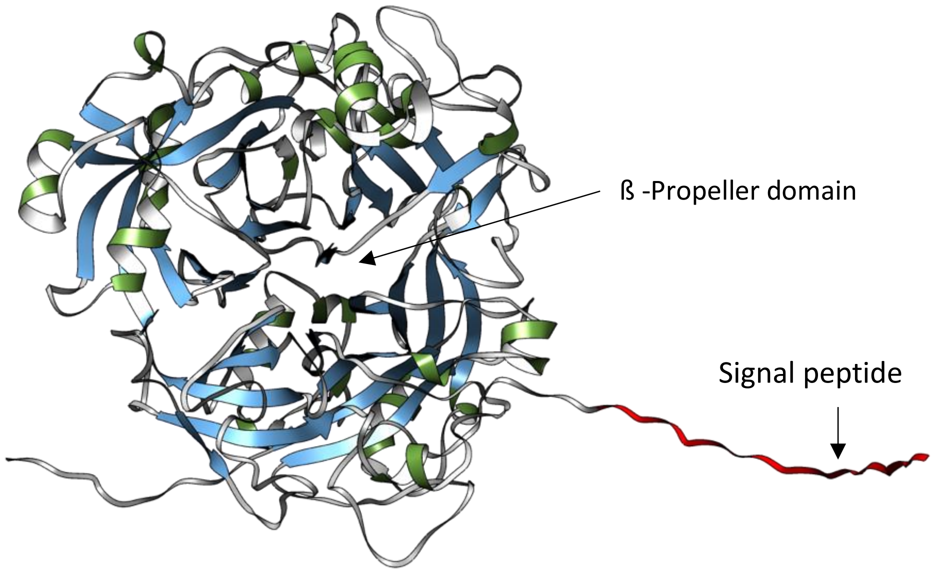

B

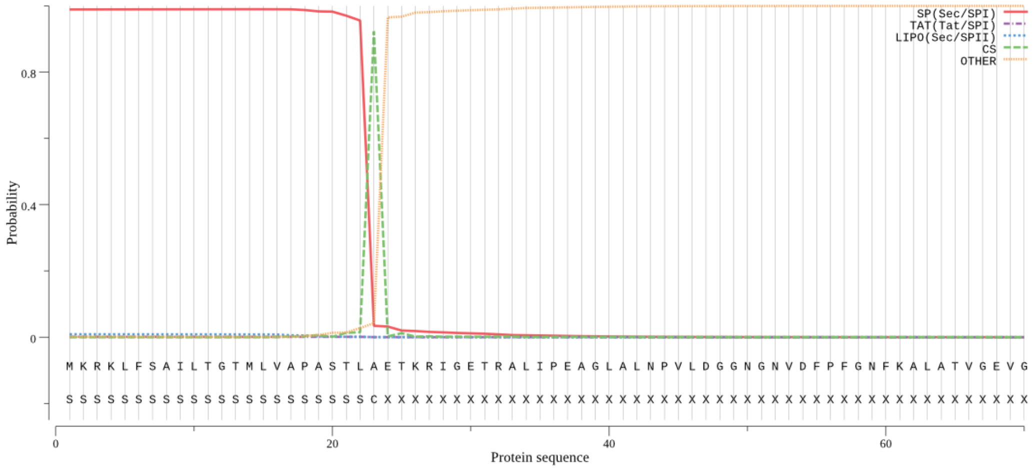

Figure S7. Alignment of Psip1 protein sequences from *Prochlorococcus* and marine *Synechococcus*. Conserved predicted active site amino acid residues are shown in colour.

```
Pro_EQPAC1_HLI_Lba|PsiP1        MKRKLFSAILTGTMLVAPASTLAETKRIGETRALIPEAGLALNP--VLDGGNGNVDFPFG    58
Pro_MED4_HLI_Lba|PsiP1          MKRKLFSAILTGTMLVAPASTLAETKRIGETRALIPEAGLALNP--VLDGGNGNVDFPFG    58
Syn_A18-40_IIIa_3dB|PsiP1       ---------------------MAKKVTIGQTTALIPEAGSAIKDEWIIDGESGNTNFPYA    39
Syn_RS9915_IIIa_3dB|PsiP1       ---------------------MAKKVTIGQTTALIPEAGSAIKDEWIIDGESGNTNFPYA    39
Syn_A15-24_IIIa_3c|PsiP1        ---------------------MAKKVTIGQTTALIPEAGSAIQPEWLLDGESGNTNFPYS    39
Syn_BOUM118_IIIa_3c|PsiP1       ---------------------MAKLVTIGQTTALIPEAGSAVQPEWLNDGQSGNINFPYS    39
Syn_A18-46.1_IIIa_3c|PsiP1      ------------------------MVTIGQTTALIPEAGSAVQPEWLNDGQSGNINFPYS    36
Syn_WH8102_IIIa_3c|PsiP1        ---------------------MAKMVTIGQTTALIPEAGSAVQPEWLNDGQSGNINFPYS    39
Syn_WH8103_IIIa_3bB|PsiP1       ------------------------LVTIGQTTALIPEAGSAVQPEWLNDGQSGNINFPYS    36
                                                           **:* ******* *::   : ** .** :**:.

Pro_EQPAC1_HLI_Lba|PsiP1        NFKALATVGEVG-DNGLALTGYPDGQAAYLLDNDTIRVVYQSESYATMG----KAPVPET    113
Pro_MED4_HLI_Lba|PsiP1          NFKALATVGEVG-DNGLALTGYPDGQAAYLLDNDTIRVVYQSESYATMG----KAPVPET    113
Syn_A18-40_IIIa_3dB|PsiP1       DFKALATVGEVGPKSRLALTGYPDGNAAWLKDDDTVRVVYQSESYGTLG----SSYDPET    95
Syn_RS9915_IIIa_3dB|PsiP1       DFKALATVGEVGPKSRLALTGYPDGNAAWLKDDDTVRVVYQSESYGTLG----SSYDPET    95
Syn_A15-24_IIIa_3c|PsiP1        SYKALATVGEVDAKTKLGLTGYPDGQAAWLVDDDTVRVAYQSESYANVTGYRTGVAEGET    99
Syn_BOUM118_IIIa_3c|PsiP1       SYKALATVGEVDADNGLGLTGYPDGQAAWLQDDDTVRVAYQSESYAHIYGRT---PAPET    96
Syn_A18-46.1_IIIa_3c|PsiP1      SYKALATVGEVDADNGLGLTGYPDGQAAWLQDDDTVRVAYQSESYAHIYGRT---PAPET    93
Syn_WH8102_IIIa_3c|PsiP1        SYKALATVGEVDADNGLGLTGYPDGQAAWLQDDDTVRVAYQSESYAHIYGRT---PAPET    96
Syn_WH8103_IIIa_3bB|PsiP1       SYKALATVGEVDADNGLGLTGYPDGQAAWLQDDDTVRVAYQSESYAHIYGRT---PAPET    93
                                .:*********. .. *.*******:**:* *:**:**.******. :          **

Pro_EQPAC1_HLI_Lba|PsiP1        YNWEMKNGVTFSGSHIHTIDYDRAKFAKFMKNGSSAEGMVKDSGKLFNTIYNVFGDEVTK    173
Pro_MED4_HLI_Lba|PsiP1          YNWEMKNGVTFSGSHIHTIDYDRAKFAKFMKNGSSAEGMVKDSGKLFNTIYNVFGDEVTK    173
Syn_A18-40_IIIa_3dB|PsiP1       WPHELESGVTFTGSKIHYIDYDRTAFADFMSSGIAASEMVKDSGILYDKAYNLFGEEVTP    155
```

```
Syn_RS9915_IIIa_3dB|PsiP1       WPHELESGVTFTGSKIHYIDYDRTAFADFMSSGIAASEMVKDSGILYDKAYNLFGEEVTP       155
Syn_A15-24_IIIa_3c|PsiP1        YARAMKTGVTFTGSRIHTIDYARDAFADFMNNDSAASDMVKGSGFLFNRVFNLFGEEVTP       159
Syn_BOUM118_IIIa_3c|PsiP1       YPQEMETGVTFSGSKIHYIDYSRDAFADFMSNDSAASDMVEGSGFLFNRVFNLFGEEVTP       156
Syn_A18-46.1_IIIa_3c|PsiP1      YPQEMETGVTFSGSKIHYIDYSRDAFADFMGNDSAASDMVEGSRFLFNRVFNLFGEEVTP       153
Syn_WH8102_IIIa_3c|PsiP1        YPQEMETGVTFSGSKIHYIDYSRDAFADFMGNDSAASDMVEGSGFLFNRVFNLFGEEVTP       156
Syn_WH8103_IIIa_3bB|PsiP1       YPQEMETGVTFSGSKIHYIDYSRDAFADFMGNDSAASDMVEGSGFLFNRVFNLFGEEVTP       153
                                :   ::.****:**:** *** *  **.** .. :*. **:.*  *::  :*:**:***

Pro_EQPAC1_HLI_Lba|PsiP1        EN-------LVWGNQALPSQRVVPFLDKYKLSEADFFLQSFCGAWYEQANKYGNGIGLED       226
Pro_MED4_HLI_Lba|PsiP1          EN-------LVWGNQALPSQRVVPFLDKYKLSEADFFLQSFCGAWYEQANKYGNGIGLED       226
Syn_A18-40_IIIa_3dB|PsiP1       KNSDPADLGAKWGNQTTPSGTVIEFEN--PLSEADFFFHSFCGAWYEPAHRYGEDIGFVD       213
Syn_RS9915_IIIa_3dB|PsiP1       KNSDPADLGAKWGNQTTPSGTVIEFEN--PLSEADFFFHSFCGAWYEPAHRYGEDIGFVD       213
Syn_A15-24_IIIa_3c|PsiP1        KNTDPTDLAAKWGNQTLPSGDIIEFSA--PLADADFFFHSFCGAWYEPANRYGEGQGFSD       217
Syn_BOUM118_IIIa_3c|PsiP1       KNTDPEDLAAKWGNQTLPSGDIVEFAS--PLSETDFYFHSFCGAWYEPANRYGEGQGFSD       214
Syn_A18-46.1_IIIa_3c|PsiP1      KNTDPEDKAAKWGNQTLPSGDIVEFAS--PLSETDFYFHSFCGAWYEPANRYGEGQGFSD       211
Syn_WH8102_IIIa_3c|PsiP1        KNTDPEDKAAKWGNQTLPSGDIVEFAS--PLSETDFYFHSFCGAWYEPANRYGEGQGFSD       214
Syn_WH8103_IIIa_3bB|PsiP1       KNTDPEDLAAKWGNQTLPSGDIVEFAS--PLSETDFYFHSFCGAWYEPANRYGEGQGFSD       211
                                :*         ****: **  :: *     *:::**:::******** *::**:. *: *

Pro_EQPAC1_HLI_Lba|PsiP1        DVWLTAEEWEIGRMFTG-SKKTGRTESDKTMGLASIAVDVKNEVAYTAPALGQTGYEKLM       285
Pro_MED4_HLI_Lba|PsiP1          DVWLTAEEWEIGRMFTG-SKKTGRTESDKTMGLASIAVDVKNEVAYTAPALGQTGYEKLM       285
Syn_A18-40_IIIa_3dB|PsiP1       DVWLTAEEWVISRAFNRADGSLGHAMANETMGLAATVTDIENSTLYSVPALGTTGYEKMM       273
Syn_RS9915_IIIa_3dB|PsiP1       DVWLTAEEWVISRAFNRADGSLGHAMANETMGLAATVTDIENSTLYSVPALGTTGYEKMM       273
Syn_A15-24_IIIa_3c|PsiP1        DIWLMAEEWDIGED-AFGP--AGSAVGNETMGLAAMAVDVANEVAYSVPALGQTGYEKIA       274
Syn_BOUM118_IIIa_3c|PsiP1       DIWLMAEEWDIGWG-NFAPGYAGEAVGNETMGLAAMAVDVANSVAYSVPALGQTGYEKIA       273
Syn_A18-46.1_IIIa_3c|PsiP1      DIWLMAEEWDIGFG-NFAPGYAGKAVGNETMGLAAMAVDVANSVAYSVPALGQTGYEKIA       270
Syn_WH8102_IIIa_3c|PsiP1        DIWLMAEEWDIGFG-NFAPGYAGKAVGNETMGLAAMAVDVANSVAYSVPALGQTGYEKIA       273
Syn_WH8103_IIIa_3bB|PsiP1       DIWLMAEEWDIGWG-NFAPGYAGEAVGNETMGLAAMAVDVANSVAYSVPALGQTGYEKIA       270
```

```
                                *:** **** *.          * : .::*****: ..*: *.. *:.**** *****:
Pro_EQPAC1_HLI_Lba|PsiP1        PINPQHKDYVVIVGAGYNHNQEPAPLKVYVGMKDRLPDGSEIDYSTANERDAFLGRNGML    345
Pro_MED4_HLI_Lba|PsiP1          PINPQHKDYVVIVGAGYNHNQEPAPLKVYVGMKDRLPDGSEIDYSTANERDAFLGRNGML    345
Syn_A18-40_IIIa_3dB|PsiP1       PLNPGTEEYQVMVMAGYNHGHEPAPLKIFIGRKGYDAEGNKIT-SDHNERDQFLGRNGLL    332
Syn_RS9915_IIIa_3dB|PsiP1       PLNPGTEEYQVMVMAGYNHGHEPAPLKIFIGRKGYDAEGNKIT-SDHNERDQFLGRNGLL    332
Syn_A15-24_IIIa_3c|PsiP1        PLNTGESDYVVMVTAGYNHGQDPAPLKIYVGRKGYDAEGNVIT-EDHSERDQFLGRNGML    333
Syn_BOUM118_IIIa_3c|PsiP1       PLNTGESDYVVMVTAGYNHGQDPAPLKIYVGRKGYDAEGNEIT-EDHSERDQFLGRNGML    332
Syn_A18-46.1_IIIa_3c|PsiP1      PLNTGESDYVVMVTAGYNHGQDPAPLKIYVGRKGYDAEGNEIT-EDHSERDQFLGRNGML    329
Syn_WH8102_IIIa_3c|PsiP1        PLNTGESDYVVMVTAGYNHGQDPAPLKIYVGRKGYDAEGNEIT-EDHSERDQFLGRNGML    332
Syn_WH8103_IIIa_3bB|PsiP1       PLNTGESDYVVMVTAGYNHGQDPAPLKIFVGRKGYDAEGNEIT-EDHSERDQFLGRNGML    329
                                *:*   .:* *:* *****.::*****:::* *.   :*. *  .  .*** ******:*
Pro_EQPAC1_HLI_Lba|PsiP1        YGRIYGFAMPTKSYASLGLEANPA------AKMMDEYLQNA-DAPNSFEGRFYPTSYQWG    398
Pro_MED4_HLI_Lba|PsiP1          YGRIYGFAMPTKSYASLGLEANPA------AKMMDEYLQNA-DAPNSFEGRFYPTSYQWG    398
Syn_A18-40_IIIa_3dB|PsiP1       WGQLYGQAVSNKTIKNLGLTNDSNGNGLFDEHIVDEYLTNA-AAPDKFKGRFYPTSFQWG    391
Syn_RS9915_IIIa_3dB|PsiP1       WGQLYGQAVSNKTIKNLGLTNDSNGNGLFDEHIVDEYLTNA-AAPDKFKGRFYPTSFQWG    391
Syn_A15-24_IIIa_3c|PsiP1        WGQLYGQTLKNKHFDKLGIVADEDGNGRFDDQVMNTYLTSQAKAGDSYKGRFYPTSFQWG    393
Syn_BOUM118_IIIa_3c|PsiP1       WGQLYGQALKNNHFEKLGIVADEDGNGVFDDQVMNTYLTSQAKAGDTFKGRFYPTSFQWG    392
Syn_A18-46.1_IIIa_3c|PsiP1      WGQLYGQALKNKHFDKLGIVADEDGNGVFDDQVMNTYLTSQAKAGDSYKGRFYPTSFQWG    389
Syn_WH8102_IIIa_3c|PsiP1        WGQLYGQALKNKHFDKLGIVADEDGNGVFDDQVMNTYLTSQAKAGDSYKGRFYPTSFQWG    392
Syn_WH8103_IIIa_3bB|PsiP1       WGQLYGQALKNKHFDKLGIVADEDGNGVFDDQVMNTYLTSQAKAGDSYKGRFYPTSFQWG    389
                                :*::** :: .:   .**:  :         :::: ** .   * :.::*******:***
Pro_EQPAC1_HLI_Lba|PsiP1        GWDNPVSVNNTEMMLWEQAGEQPEG--YTFFNGDSKAEHPAVDPDINRTRYVQNMTNKGG    456
Pro_MED4_HLI_Lba|PsiP1          GWDNPVSVNNTEMMLWEQAGEQPEG--YTFFNGDSKAEHPAVDPDINRTRYVQNMTNKGG    456
Syn_A18-40_IIIa_3dB|PsiP1       GWDEPTAVGDQEMYLWERPEEQPNNGKYKFFQGDAKTEHPAVDPS-GKARWFQNMTDEGA    450
Syn_RS9915_IIIa_3dB|PsiP1       GWDEPTAVGDQEMYLWERPEEQPNNGKYKFFQGDAKTEHPAVDPS-GKARWFQNMTDEGA    450
Syn_A15-24_IIIa_3c|PsiP1        GWDEPTAVGNTEMFLWERPEEQPKN--YTFFQGDKKTEHQAIDPS-GKARWFQNMTDEGA    450
```

```
Syn_BOUM118_IIIa_3c|PsiP1       GWDEPTAVGNTEMFLWERPEEQPKN--YTFFQGDKKTEHQAIDPS-GKARWFQNMTDEGA    449
Syn_A18-46.1_IIIa_3c|PsiP1      GWDEPTAVGNTEMFLWERPEEQPKN--YTFFNGDKKTEHQAIDPS-GKARWFQNMTDEGA    446
Syn_WH8102_IIIa_3c|PsiP1        GWDEPTAVGNTEMFLWERPEEQPKN--YTFFNGDKKTEHQAIDPS-GKARWFQNMTDEGA    449
Syn_WH8103_IIIa_3bB|PsiP1       GWDEPTAVGNTEMFLWERPEQQPKN--YTFFQGDKKTEHQAIDPS-GKARWFQNMTDEGA    446
                                ***:*.:*.: ** ***:  :**:.  *.**:** *:** *:**. .::*:.****::*.

Pro_EQPAC1_HLI_Lba|PsiP1        ILGFDFGFIGATLD----AADGDLPEFLPATGIRVVPAIDGALTLKTGGEGVVKDGS---    509
Pro_MED4_HLI_Lba|PsiP1          ILGFDFGFIGATLD----AADGDLPEFLPATGIRVVPAIDGALTLKTGGEGVVKDGS---    509
Syn_A18-40_IIIa_3dB|PsiP1       LMGINIKNIAKQLKNNPDDDGNLLPDFLNYKAVRTVAAVDGALTIDVGDEGLAHAGSLNP    510
Syn_RS9915_IIIa_3dB|PsiP1       LMGINIKNIAKQLKNNPDDDGNLLPDFLNYKAVRTVAAVDGALTIDVGDEGLAHAGSLNP    510
Syn_A15-24_IIIa_3c|PsiP1        LLGFNLKNLAKQLKSNPDADGNSLPDYINYKSVVTIPATDGSLRVDVGDEGLAHKGEANP    510
Syn_BOUM118_IIIa_3c|PsiP1       LLGFNLKNLAKQLNNNPDADGNLLPDYLNYKSVVTIPATDGSLRVDVGDEGLAHKGEANP    509
Syn_A18-46.1_IIIa_3c|PsiP1      LLGFDLKNLAKQLKSNPDADGNLLPDYLNYKSVVTIPATDGSLRVDVGDEGLAHKGEANP    506
Syn_WH8102_IIIa_3c|PsiP1        LLGFDLKNLAKQLKSNPDADGNLLPDYLNYKSVVTIPATDGSLRVDVGDEGLAHKGEANP    509
Syn_WH8103_IIIa_3bB|PsiP1       LLGFNLKNLAKQLKSNPDADGNLLPDYINYKSVVTIPATDGSLRVDVGDEGLAHKGEANP    506
                                ::*:::  :.  *.      .. **:::  ..: .: * **:* :..*.**:.: *.

Pro_EQPAC1_HLI_Lba|PsiP1        -----AAIHVEKNKAAMVSPDGLYWTKHADG-SYLIVDEDSGNDFGERKYVLPIKESDMT    563
Pro_MED4_HLI_Lba|PsiP1          -----AAIHVEKNKAAMVSPDGLYWTKHADG-SYLIVDEDSGNDFGERKYVLPIKESDMT    563
Syn_A18-40_IIIa_3dB|PsiP1       DGNLTASMHVEKQVNKMVAPDGLYWAKGSDGPGVLIVDEDSGNDYGERKYALPINN-KME    569
Syn_RS9915_IIIa_3dB|PsiP1       DGNLTASMHVEKQVNKMVAPDGLYWAKGSDGPGVLIVDEDSGNDYGERKYALPINN-KME    569
Syn_A15-24_IIIa_3c|PsiP1        DGSLTHATHVEKGVEKIVSNDGLYWAKGKDG-NVLILDEDSGNDYGERKIALPLKG--ME    567
Syn_BOUM118_IIIa_3c|PsiP1       DGSLTHAIHVEKGVEKIVANDGLYWAKGKGG-NVLILDEDSGNDYGERKIALPLKG--ME    566
Syn_A18-46.1_IIIa_3c|PsiP1      DGSLTHAIHVEKGVEKIVANDGLYWAKGKGG-NVLILDEDSGNDYGERKIALPIKG-NMQ    564
Syn_WH8102_IIIa_3c|PsiP1        DGSLTHAIHVEKGVEKIVANDGLYWAKGKGG-NVLILDEDSGNDYGERKIALPIKR-NMQ    567
Syn_WH8103_IIIa_3bB|PsiP1       DGSLTHAIHVEKGVEKIVANDGLYWAKGKGG-NVLILDEDSGNDYGERKIALPVKG-NME    564
                                      : ****    :*: *****:*  .* . **:*******:**** .**::   *

Pro_EQPAC1_HLI_Lba|PsiP1        LSEPNTGYLLGLAGGKHSSRYEAGASALGGAFSKA------TTSEFSGSWNVTALTTKKS    617
```

```
Pro_MED4_HLI_Lba|PsiP1         LSEPNTGYLLGLAGGKHSSRYEAGASALGGAFSKA------TTSEFSGSWNVTALTTKKS      617
Syn_A18-40_IIIa_3dB|PsiP1      LRDPATGYLLATAGGKLNPRQIAGAAALPGSSWLPEDGNGGQGAEFSGSWDITGLVAKKD      629
Syn_RS9915_IIIa_3dB|PsiP1      LRDPATGYLLATAGGKLNPRQIAGAAALPGSSWLPEDGNGGQGAEFSGSWDITGLVAKKD      629
Syn_A15-24_IIIa_3c|PsiP1       LRDEATGYFLGAAGGTLSPRYEAGAAALAGAFSAA------GTNEFSGSWDVTGMVTRKD      621
Syn_BOUM118_IIIa_3c|PsiP1      LRDEATGYFLGAAGGTLSPRYLAGATALAGAIDKP------GTNEYSGSWDVTGLVTRKD      620
Syn_A18-46.1_IIIa_3c|PsiP1     LRDEATGYFLGAAGGTLSPRYLAGATALAGAIDKP------GTNEYSGSWDVTGMVTRKD      618
Syn_WH8102_IIIa_3c|PsiP1       LRDEATGYFLGAAGGTLSPRYLAGATALAGAIDKP------GTNEYSGSWDVTGMVTRKD      621
Syn_WH8103_IIIa_3bB|PsiP1      LRDEATGYFLGAAGGTLSPRYLAGATALAGAIDKP------GTNEYSGSWDVTGLVTRKD      618
                               * :  ***:*. ***. . *  ***:** *:             *:****::*.:.::*.

Pro_EQPAC1_HLI_Lba|PsiP1       NGLFGSKFYSMDELAGTGEQEIIQGIDTDDQLFIGVVQARGESGGAVAEEGGDAGGQVFQ      677
Pro_MED4_HLI_Lba|PsiP1         NGLFGSKFYSMDELAGTGEQEIIQGIDTDDQLFIGVVQARGESGGAVAEEGGDAGGQVFQ      677
Syn_A18-40_IIIa_3dB|PsiP1      DGS----FYSKEELSGTGLDSIQNAIPIEDHLYQGVVQYRGESGGQVYEEQADAGGQIFQ      685
Syn_RS9915_IIIa_3dB|PsiP1      DGS----FYSKEELAGTGLDSIQNAIPIEDHLYQGVVQYRGESGGQVYEEQADAGGQIFQ      685
Syn_A15-24_IIIa_3c|PsiP1       DGS----FYSKEELSGSGMQDVADMVHIEDHTYIGVVQARPESGGQVEEIGGDAGGQVFM      677
Syn_BOUM118_IIIa_3c|PsiP1      DGS----FYSKEELSGSGMQDVADMVHIEDHTYVGVVQARPESGGQVEEISGDAGGQVFM      676
Syn_A18-46.1_IIIa_3c|PsiP1     DGS----FYSKEELSGSGMQDVADLVHIEDHTYIGVVQARPESGGQVEEISGDAGGQVFM      674
Syn_WH8102_IIIa_3c|PsiP1       DGS----FYSKEELSGSGMQDVADLVHIEDHTYIGVVQARPESGGQVEEISGDAGGQVFM      677
Syn_WH8103_IIIa_3bB|PsiP1      DGS----FYSKEELSGSGMQDVADMVHIEDHTYVGVVQARPESGGQVEEISGDAGGQVFM      674
                               :*     *** :**:*:* :.: : :  :*: : ***: * **** * *  .*****:*

Pro_EQPAC1_HLI_Lba|PsiP1       FTFNLPEKSEGENIAQF*    694
Pro_MED4_HLI_Lba|PsiP1         FTFNLPEKSEGENIAQF*    694
Syn_A18-40_IIIa_3dB|PsiP1      FTMSDFF*----------    692
Syn_RS9915_IIIa_3dB|PsiP1      FTMSDFF*----------    692
Syn_A15-24_IIIa_3c|PsiP1       FEMSNFY*----------    684
Syn_BOUM118_IIIa_3c|PsiP1      FEMNGFF*----------    683
Syn_A18-46.1_IIIa_3c|PsiP1     FEMNGFF*----------    681
```

```
Syn_WH8102_IIIa_3c|PsiP1        FEMNGFF*-----------   684
Syn_WH8103_IIIa_3bB|PsiP1       FEMNGFF*-----------   681
                                * :.
```

Figure S8. Non-metric multidimensional scaling of nutrient stress genes across TARA stations. Nutrient stress genes are identical to those used by Ustick et al., 2021 (17) with the addition of the relative abundance of *psip1*. nMDS is based on Kulczynski dissimilarity between gene abundances. 1000 iterations were performed with a random starting configuration. Genes are colored using the nutrient stress categories defined by Ustick et al.

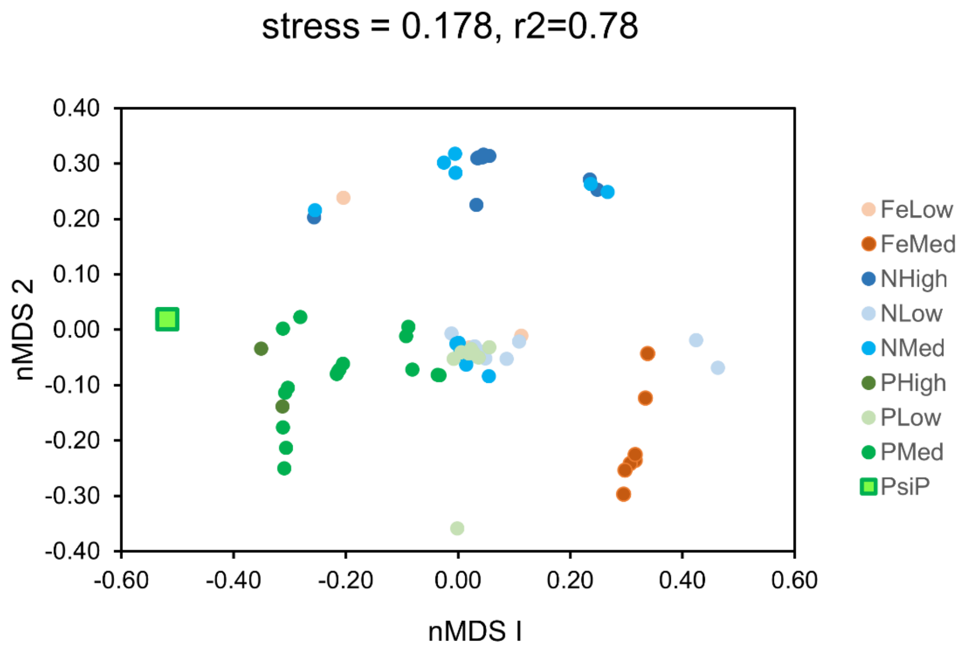

Figure S9. Phylogenetic tree of prokaryotic BLASTP sequences (E-value cut-off 10^{-20}) and Tara Ocean sequences retrieved using a HMM model of Psip1. *psip1* expression from TARA metatranscriptomes is shown in the bar chart normalized by site to the expression of ten single copy housekeeping genes (18).

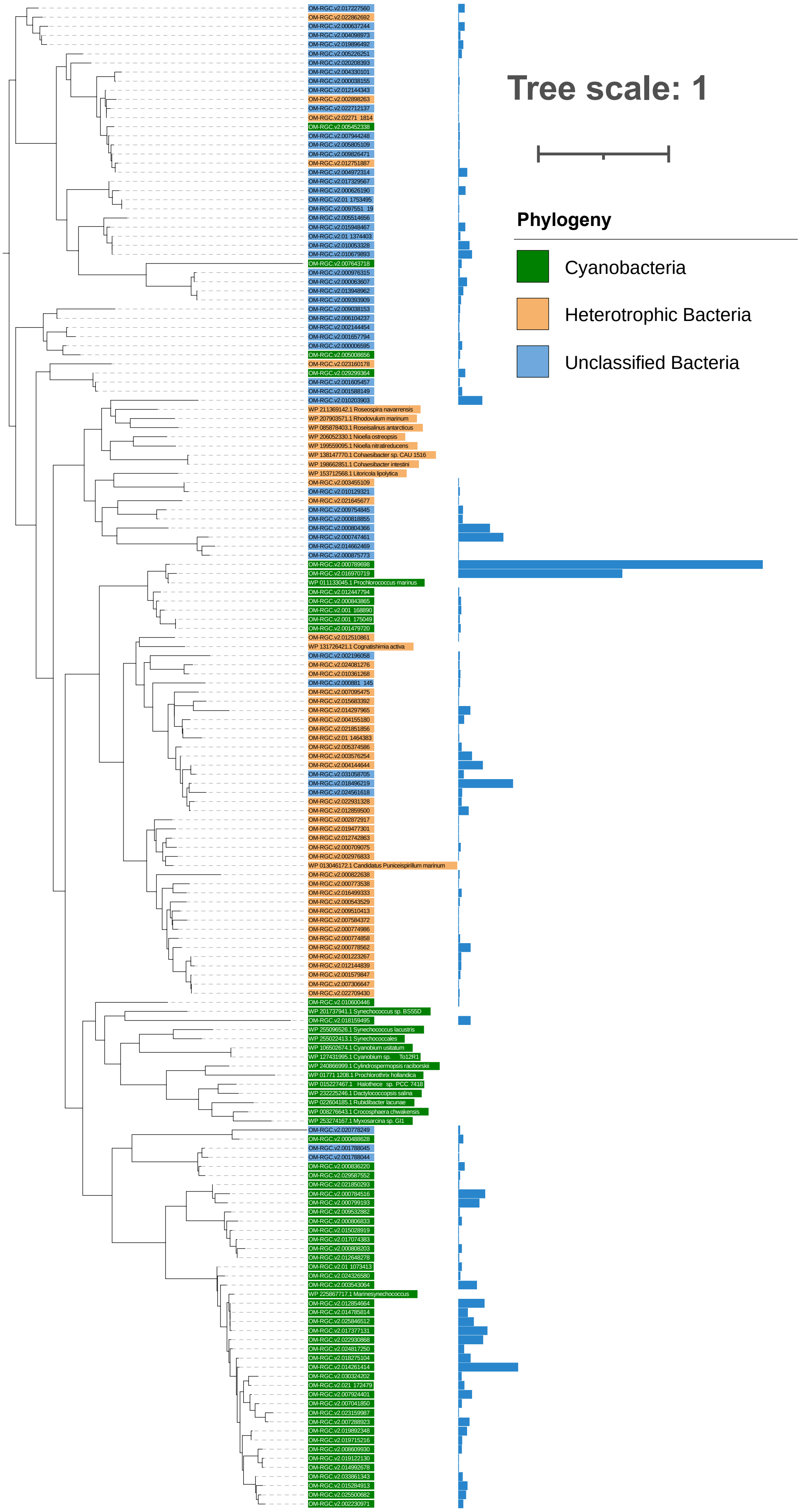

Tree scale: 1
Phylogeny
Cyanobacteria
Heterotrophic Bacteria
Unclassified Bacteria

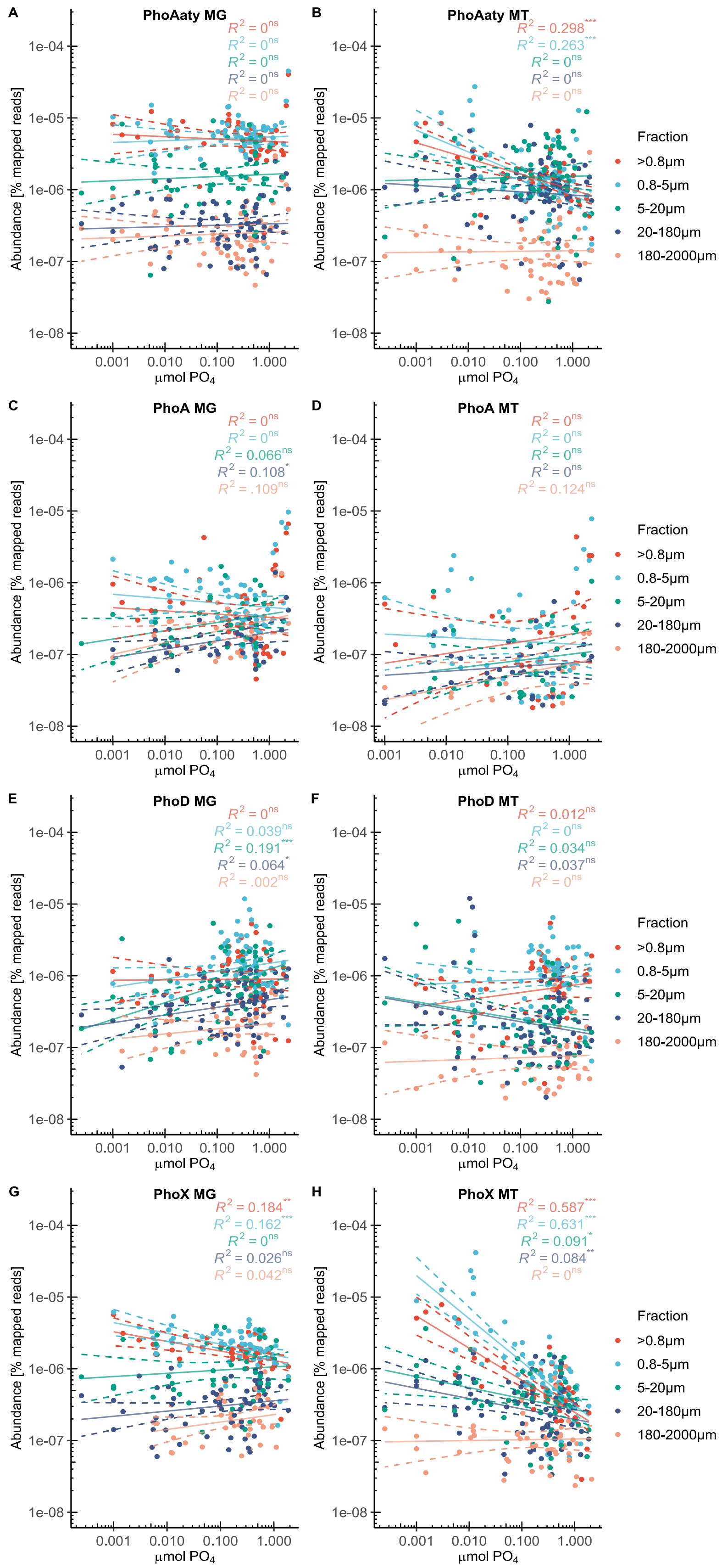

Figure S10. Correlation between the abundance of specific eukaryotic alkaline phosphatases i.e. the atypical *phoA* (phoAty) (A, B), *phoA* (C, D)*, phoD* (E, F) and *phoX* (G, H) with inorganic phosphate (PO_4) concentrations in the metagenome (A, C, E, G) and metatranscriptome (B, D, F, H) of the Marine Atlas Tara Ocean Unigenes (MATOU) database. Abundance values are split according to the eukaryote size fraction applied prior to nucleic acid extraction. Linear regressions of log_{10} transformed abundance vs PO_4 concentration are shown (solid lines) together with 95% confidence intervals (dashed lines). R^2 values are shown in the corresponding colour. * = p<0.05, ** = p<0.01, *** = p<0.001, ns = not significant.

Figure S11. Phylogeny of Psip1 homologs from eukaryotic size fractions of the Marine Atlas *Tara Oceans* Unigenes (MATOU) database, together with eukaryote reference sequences. The MATOU assigned phylogeny of homologs is shown by colour, and transcript abundance of sequences, normalized across all ocean sites and size fractions, is split by size fraction. Normalization was based on the percentage of mapped reads.

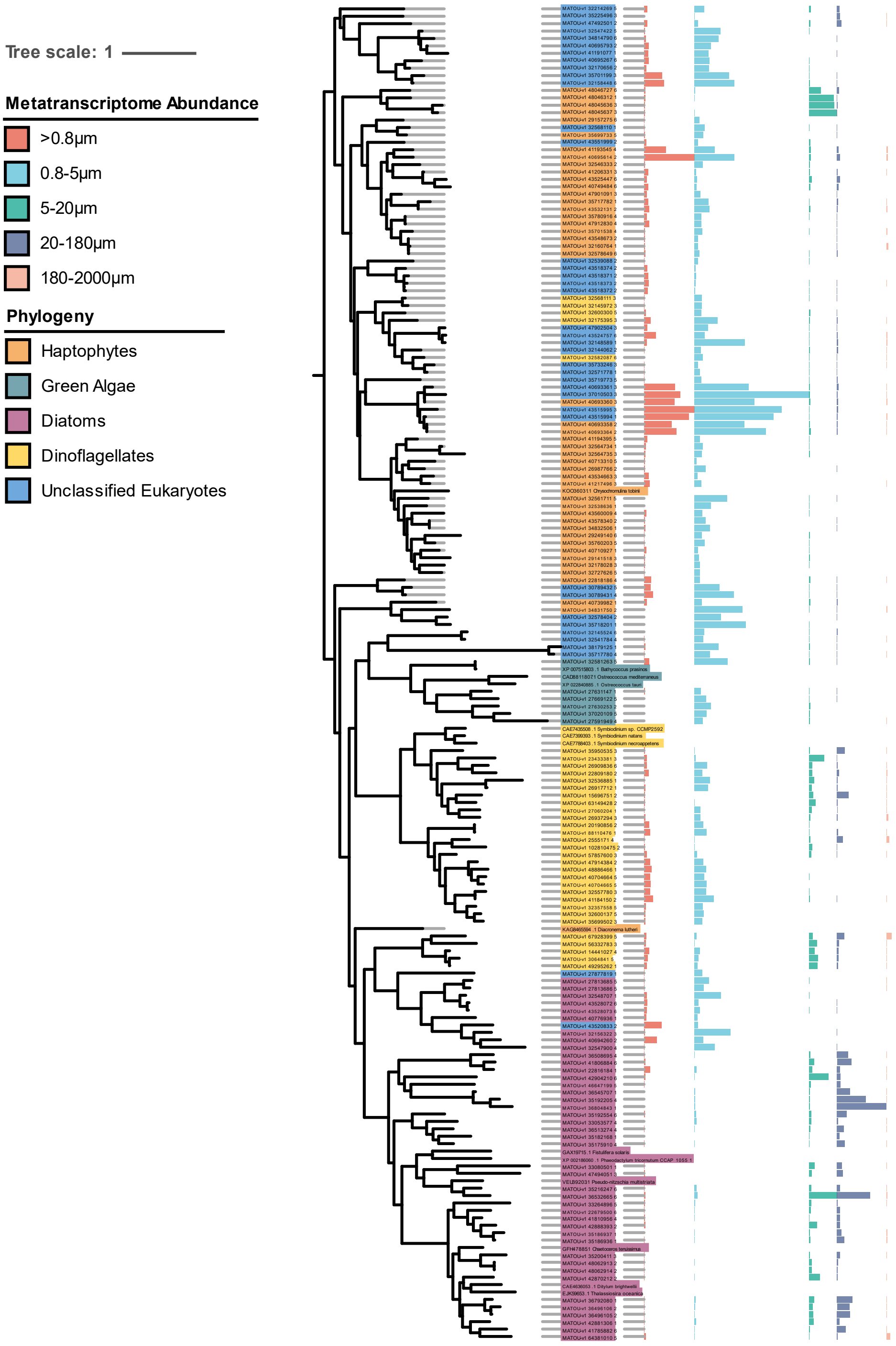

References

1. D. H. Lee, *et al*., A novel psychrophilic alkaline phosphatase from the metagenome of tidal flat sediments. *BMC Biotech* **15**, 1-13 (2015).
2. R. Zaheer, R. Morton, M. Proudfoot, A. Yakunin, T. M. Finan, Genetic and biochemical properties of an alkaline phosphatase PhoX family protein found in many bacteria. *Environ Microbiol* **11**, 1572–1587 (2009).
3. L. M. Westermann, *et al.*, Bacterial catabolism of membrane phospholipids links marine biogeochemical cycles. *Sci Adv* **9**, eadf5122 (2023).
4. N. K. Roy, R. K. Ghosh, J. Das, Monomeric alkaline phosphatase of *Vibrio cholerae*. *J Bacteriol* **150**, 1033–1039 (1982).
5. J. R. Wu, *et al.*, Cloning of the gene and characterization of the enzymatic properties of the monomeric alkaline phosphatase (PhoX) from *Pasteurella multocida* strain X-73. *FEMS Microbiol Lett* **267**, 113–120 (2007).
6. M. Ishibashi, S. Yamashita, M. Tokunaga, Characterization of halophilic alkaline phosphatase from *Halomonas* sp. 593, a moderately halophilic bacterium. *Biosci, Biotech Biochem* **69,** 1213-1216 (2005).
7. S. Zappa, J. L Rolland, D. Flament, Y. Gueguen, J. Boudrant, J. Dietrich, Characterization of a highly thermostable alkaline phosphatase from the euryarchaeon *Pyrococcus abyssi*. *Appl Environ Microbiol* **67**, 4504-4511 (2001).
8. Y. Suzuki, Y. Mizutani, T. Tsuji, N. Ohtani, K. Takano, M. Haruki, M. Morikawa, S. Kanaya, Gene cloning, overproduction, and characterization of thermolabile alkaline phosphatase from a psychrotrophic bacterium. *Biosci, Biotech Biochem* **69,** 364-373 (2005).
9. R. S. Moura, J. F. Martín, A. Martín, P. Liras, Substrate analysis and molecular cloning of the extracellular alkaline phosphatase of *Streptomyces griseus*. *Microbiology* **147,** 1525-1533 (2001).
10. C. L. Wojciechowski, J. P. Cardia, E. R. Kantrowitz, Alkaline phosphatase from the hyperthermophilic bacterium *T. maritima* requires cobalt for activity. *Protein Sci* **11,** 903-911 (2002).
11. J. B. Hauksson, Ó. S. Andrésson, B. Ásgeirsson, Heat-labile bacterial alkaline phosphatase from a marine *Vibrio* sp. *Enzyme Microb Technol* **27**, 66-73 (2000).
12. A. Srivastava, *et al.*, Enzyme promiscuity in natural environments: alkaline phosphatase in the ocean. *ISME J* **15**, 3375–3383 (2021).

13. H. Kobori, C. W. Sullivan, H. Shizuya, Heat-labile alkaline phosphatase from Antarctic bacteria: rapid 5′ end-labeling of nucleic acids. *Proc Natl Acad Sci USA* **81**, 6691-6695 (1984).
14. H. Kageyama, *et al.*, An alkaline phosphatase/phosphodiesterase, PhoD, induced by salt stress and secreted out of the cells of *Aphanothece halophytica*, a halotolerant cyanobacterium. *Appl Environ Microbiol* **77**, 5178–5183 (2011).
15. Y. Noskova, G. Likhatskaya, N. Terentieva, O. Son, L. Tekutyeva, L. Balabanova, A novel alkaline phosphatase/phosphodiesterase, CamPhoD, from marine bacterium *Cobetia amphilecti* KMM 296. *Mar Drugs* **17**, 657 (2019).
16. P. Virtanen, *et al.*, SciPy 1.0: fundamental algorithms for scientific computing in Python. *Nat Methods* **17**, 261–272 (2020).
17. L. J. Ustick, *et al.*, Metagenomic analysis reveals global-scale patterns of ocean nutrient limitation. *Science* **372**, 287–291 (2021).
18. A. Milanese, *et al.*, Microbial abundance, activity and population genomic profiling with mOTUs2. *Nat Comms* **10**, 1014 (2019).
